# High frequencies of alpha common cold coronavirus/SARS-CoV-2 cross-reactive functional CD4^+^ and CD8^+^ memory T cells are associated with protection from symptomatic and fatal SARS-CoV-2 infections in unvaccinated COVID-19 patients

**DOI:** 10.3389/fimmu.2024.1343716

**Published:** 2024-03-28

**Authors:** Pierre-Gregoire Coulon, Swayam Prakash, Nisha R. Dhanushkodi, Ruchi Srivastava, Latifa Zayou, Delia F. Tifrea, Robert A. Edwards, Cesar J. Figueroa, Sebastian D. Schubl, Lanny Hsieh, Anthony B. Nesburn, Baruch D. Kuppermann, Elmostafa Bahraoui, Hawa Vahed, Daniel Gil, Trevor M. Jones, Jeffrey B. Ulmer, Lbachir BenMohamed

**Affiliations:** ^1^ Laboratory of Cellular and Molecular Immunology, Gavin Herbert Eye Institute, University of California Irvine, School of Medicine, Irvine, CA, United States; ^2^ Department of Pathology and Laboratory Medicine, School of Medicine, University of California Irvine, Irvine, CA, United States; ^3^ Department of Surgery, Divisions of Trauma, Burns and Critical Care, School of Medicine, University of California Irvine, Irvine, CA, United States; ^4^ Department of Medicine, Division of Infectious Diseases and Hospitalist Program, School of Medicine, University of California Irvine, Irvine, CA, United States; ^5^ Université Paul Sabatier, Infinity, Inserm, Toulouse, France; ^6^ Department of Vaccines and Immunotherapies, TechImmune, LLC, University Lab Partners, Irvine, CA, United States; ^7^ Institute for Immunology, The University of California Irvine, School of Medicine, Irvine, CA, United States

**Keywords:** SARS-CoV-2, symptomatic, asymptomatic, COVID-19, common cold coronavirus, CD4 + T cells, CD8 + T cells, exhaustion α-CCCs/SARS-CoV-2 cross-reactive T cells in asymptomatic COVID-19 infection

## Abstract

**Background:**

Cross-reactive SARS-CoV-2-specific memory CD4^+^ and CD8^+^ T cells are present in up to 50% of unexposed, pre-pandemic, healthy individuals (UPPHIs). However, the characteristics of cross-reactive memory CD4^+^ and CD8^+^ T cells associated with subsequent protection of asymptomatic coronavirus disease 2019 (COVID-19) patients (i.e., unvaccinated individuals who never develop any COVID-19 symptoms despite being infected with SARS-CoV-2) remains to be fully elucidated.

**Methods:**

This study compares the antigen specificity, frequency, phenotype, and function of cross-reactive memory CD4^+^ and CD8^+^ T cells between common cold coronaviruses (CCCs) and SARS-CoV-2. T-cell responses against genome-wide conserved epitopes were studied early in the disease course in a cohort of 147 unvaccinated COVID-19 patients who were divided into six groups based on the severity of their symptoms.

**Results:**

Compared to severely ill COVID-19 patients and patients with fatal COVID-19 outcomes, the asymptomatic COVID-19 patients displayed significantly: (i) higher rates of co-infection with the 229E alpha species of CCCs (α-CCC-229E); (ii) higher frequencies of cross-reactive functional CD134^+^CD137^+^CD4^+^ and CD134^+^CD137^+^CD8^+^ T cells that cross-recognized conserved epitopes from α-CCCs and SARS-CoV-2 structural, non-structural, and accessory proteins; and (iii) lower frequencies of CCCs/SARS-CoV-2 cross-reactive exhausted PD-1^+^TIM3^+^TIGIT^+^CTLA4^+^CD4^+^ and PD-1^+^TIM3^+^TIGIT^+^CTLA4^+^CD8^+^ T cells, detected both *ex vivo* and *in vitro*.

**Conclusions:**

These findings (i) support a crucial role of functional, poly-antigenic α-CCCs/SARS-CoV-2 cross-reactive memory CD4^+^ and CD8^+^ T cells, induced following previous CCCs seasonal exposures, in protection against subsequent severe COVID-19 disease and (ii) provide critical insights into developing broadly protective, multi-antigen, CD4^+^, and CD8^+^ T-cell-based, universal pan-Coronavirus vaccines capable of conferring cross-species protection.

## Introduction

The coronavirus disease 2019 (COVID-19) pandemic has created one of the largest global health crises in nearly a century (1–3). As of February 2024, the COVID-19 outbreak has affected over 700 million people worldwide, with the number of deaths directly related to severe symptomatic COVID-19 infections reaching 7 million worldwide (1, 2, 4). Some unvaccinated symptomatic COVID-19 patients produce severe symptoms that typically begin with mild upper respiratory syndrome but may further develop into severe respiratory distress and death, particularly in immunocompromised individuals and those with pre-existing co-morbidities (5–8). In contrast, other unvaccinated individuals never develop any COVID-19 symptoms despite being infected with SARS-CoV-2 (5, 9, 10). The underlying mechanisms that lead to protection from symptomatic and fatal SARS-CoV-2 infection in unvaccinated COVID-19 patients remain to be fully elucidated.

There is a growing body of evidence in support of the important role that T-cell responses in protection against COVID-19, as recently reviewed by Wherry and Barouch (11): (i) cross-reactive poly-antigenic CD4^+^ and CD8^+^ T-cell responses in COVID-19 patients appear to contribute to the resolution of SARS-CoV-2 infection and reduction in severe symptoms (12–22); (ii) SARS-CoV-2-specific CD4^+^ and CD8^+^ T-cell responses reduced viral loads in non-human primates (23, 24); (iii) SARS-CoV-2-infected patients with agammaglobulinemia and B-cell depletion displayed only a small increase in COVID-19 symptoms, indicating that the cross-reactive T cells alone may have protected from severe disease (25–30); and (iv) cancer patients with B-cell deficiencies experience milder COVID-19 disease that correlated with strong SARS-CoV-2-specific CD8^+^ T-cell responses (22). Conversely, other reports have associated cross-reactive memory CD4^+^ and CD8^+^ T cells with poor COVID-19 disease outcomes (16, 31–36). However, the antigen specificity, frequency, phenotype, and function of cross-reactive memory CD4^+^ and CD8^+^ T cells that protect against the severity of COVID-19 in unvaccinated asymptomatic patients remain to be determined.

Characterizing the CCCs/SARS-CoV-2 cross-reactive memory CD4^+^ and CD8^+^ T cells in unvaccinated COVID-19 patients is a difficult task today because over 85% of adults are currently vaccinated (37–40). Nevertheless, a few studies from our group and others have detected cross-reactive CD4^+^ and CD8^+^ T cells, directed toward specific sets of conserved SARS-CoV-2 epitopes, not only from unvaccinated COVID-19 patients but also from a significant proportion (~50%) of unexposed pre-pandemic healthy individuals (UPPHI) who were never exposed to SARS-CoV-2 (1, 16, 18, 20–22, 32, 33, 41–44). Moreover, pre-existing CCCs/SARS-CoV-2 cross-reactive memory CD4^+^ and CD8^+^ T cells are also present in unvaccinated UPPHI, suggesting clones of memory T cells induced following previous exposures with seasonal CCCs (1, 16, 21, 31, 41, 43–50). However, it is not yet known whether these cross-reactive memory CD4^+^ and CD8^+^ T cells (i) preferentially cross-recognize the alpha CCCs (i.e., α-CCC-229E and α-CCC-NL63) or the beta CCCs (i.e., β-CCC-HKU1 and β-CCC-OC43) and (ii) the antigen specificity, frequency, phenotype, and function of the cross-reactive memory CD4^+^ and CD8^+^ T cells associated with protection against COVID-19 severity in unvaccinated asymptomatic patients.

In this study, we hypothesized that different clonal repertoire of CCCs/SARS-CoV-2 cross-reactive memory CD4^+^ and CD8^+^ T cells are induced by previous exposures to seasonal alpha CCCs (i.e., α-CCC-229E and α-CCC-NL63) and beta CCCs (i.e., β-CCC-HKU1 and β-CCC-OC43) and that certain clones of T cells are associated with either protective or pathogenic outcomes in SARS-CoV-2 infection. We report that, compared with unvaccinated severely ill COVID-19 patients and unvaccinated patients with fatal COVID-19 outcomes, unvaccinated asymptomatic COVID-19 patients displayed significantly (i) higher rate of the α-CCC species 229E (α-CCC-229E); (ii) higher frequencies of functional memory CD134^+^CD137^+^CD4^+^ and CD134^+^CD137^+^CD8^+^ T cells directed toward cross-reactive α-CCCs/SARS-CoV-2 epitopes from structural, non-structural, and accessory proteins; and (iii) lower frequencies of cross-reactive exhausted PD-1^+^TIM3^+^TIGIT^+^CTLA4^+^CD4^+^ and PD-1^+^TIM3^+^TIGIT^+^CTLA4^+^CD8^+^ T cells. These findings (i) support the crucial role of functional, poly-antigenic α-CCCs/SARS-CoV-2 cross-reactive memory CD4^+^ and CD8^+^ T cells, induced following previous exposures to α-CCC species, in protection against subsequent severe disease caused by SARS-CoV-2 infection and (ii) provides a strong rationale for the development of broadly protective, T-cell-based, multi-antigen universal pan-Coronavirus vaccines.

## Materials and methods

### Human study population cohort and HLA genotyping

Between July 2020 to November 2022, 600 patients were enrolled at the University of California Irvine Medical Center with various severity of COVID-19 disease under an approved Institutional Review Board–approved protocol (IRB No. 2020-5779). Written informed consent was obtained from participants before inclusion. SARS-CoV-2 positivity was defined by a positive RT-PCR on a respiratory tract sample. None of the patients enrolled in this study received any COVID-19 vaccine.

### Patient selection based on HLA-A*02:01 and HLA-DRB1*01:01 alleles

We genotyped all the 600 patients enrolled in our study for class I HLA-A*02:01 and class II HLA-DRB1*01:01 by PCR. Out of the 600 COVID-19 patients, 147 patients were positive for HLA-A*02:01 and/or HLA-DRB1*01:01 and were considered in this study (**Supplementary Figure 1**). The 147 patients were from mixed ethnicities (Hispanic (28%), Hispanic Latino (22%), Asian (16%), Caucasian (13%), mixed Afro-American and Hispanic (8%), Afro-American (5%), mixed Afro-American and Caucasian (2%), and Native Hawaiian and Other Pacific Islander descent (1%). Six percent of the patients did not reveal their race/ethnicity. The detailed demographic and clinical data for the 147 patients enrolled in this study are shown in **Table 1**.

**Table 1:** Demographic, age, HLA-genotyping, clinical parameters, and prevalence of comorbidities in unvaccinated COVID-19 patients with various degrees of disease severity.

	Patients’ characteristics classified by severity of COVID-19 (n=147)	Severity 5 (SYMP) patients died) (*n* = 26)	Severity 4 (SYMP) (ICU + vent.) (*n* = 15)	Severity 3 (SYMP) (ICU) (n = 21)	Severity 2 (SYMP) (inpatients, Reg. Adm.) (n = 64)	Severity 1 (SYMP) (ED) (n = 12)	Severity 0 (ASYMP) (n = 9)
**Demographic features**	**Age median**	65 (39–90)	52 (33–85)	53 (26–86)	57 (23–85)	51 (27–91)	27 (19–51)
	**Gender (male/female)**	19/7 (73%/27%)	9/6 (60%/40%)	13/8 (62%/38%)	37/27 (58%/42%)	5/7 (42%/58%)	5/4 (56%/44%)
**Class I & II HLA status**	**Race (% White/non-White)**	6/20 (23%/77%)	8/7 (53%/47%)	13/8 (62%/38%)	25/39 (39%/61%)	7/5 (58%/42%)	2/7 (29%/71%)
	HLA-A*0201^+^	13 (50%)	8 (53%)	12 (57%)	24 (38%)	7 (58%)	7 (78%)
**Clinical parameters**	HLA-DRB1*01:01^+^	14 (54%)	11 (73%)	12 (57%)	41 (64%)	7 (58%)	7 (78%)
	Days between onset of symptoms and blood draw (mean)	5.9	5.7	4.6	4.5	4.1	–
	Fever (>38°C)	21 (81%)	11 (73%)	10 (48%)	30 (47%)	4 (33%)	0 (0%)
	Cough	23 (88%)	13 (87%)	16 (76%)	22 (34%)	4 (33%)	0 (0%)
	Shortness of breath/dyspnea	28 (100%)	15 (100%)	6 (29%)	11 (17%)	1 (8%)	0 (0%)
	Fatigue/myalgia	9 (35%)	5 (33%)	6 (29%)	3 (5%)	3 (25%)	0 (0%)
	Headache	5 (19%)	1 (%)	4 (19%)	12 (19%)	4 (33%)	0 (0%)
	Nausea	3 (12%)	3 (20%)	3 (14%)	3 (5%)	0 (0%)	0 (0%)
	Diarrhea	7 (27%)	2 (13%)	2 (10%)	8 (13%)	0 (0%)	0 (0%)
	Anosmia/ageusia	6 (23%)	4 (27%)	6 (29%)	17 (27%)	1 (8%)	0 (0%)
	Sore throat	4 (15%)	1 (7%)	1 (5%)	3 (5%)	1 (8%)	0 (0%)
	ICU admission	26 (100%)	15 (100%)	21 (100%)	0 (0%)	0 (0%)	0 (0%)
	Ventilator support	26 (100%)	15 (100%)	0 (0%)	0 (0%)	0 (0%)	0 (0%)
	White blood cells (count: 10^3^ cells/µL of blood) (average)	14.3	10.8	10.1	8.4	6.2	8.0
**Comorbidities**	Lymphocytes (10^3^ cells/µL of blood and %) (average)	0.7 (6%)	0.9 (10%)	1.0 (13%)	1.4 (16%)	1.6 (27%)	2.4 (29.3%)
	Average number of all comorbidities	3.5	2.9	2.8	1.9	1.6	0.7
	Diabetes	14 (54%)	9 (60%)	13 (62%)	29 (45%)	4 (33%)	0 (0%)
	Hypertension (HTN)	16 (62%)	6 (40%)	9 (43%)	18 (28%)	4 (33%)	1 (11%)
	Cardiovascular disease (CVD)	17 (65%)	6 (40%)	6 (29%)	13 (20%)	3 (25%)	0 (0%)
	Coronary artery disease (CAD)	12 (46%)	5 (33%)	7 (33%)	12 (19%)	2 (17%)	0 (0%)
	Kidney diseases (CKD/ESRD)	7 (27%)	4 (27%)	6 (29%)	7 (11%)	1 (8%)	0 (0%)
	Asthma/COPD	9 (35%)	1 (7%)	3 (14%)	12 (19%)	0 (0%)	1 (11%)
	Obesity	12 (46%)	12 (80%)	7 (33%)	29 (45%)	4 (33%)	4 (44%)
	Cancer	4(15%)	0(0%)	2(10%)	6(9%)	1(8%)	0 (0%)

Unvaccinated patients (n = 147) were scored on a scale of 0–5 based on the severity of COVID-19 symptoms, regular hospital admission, intensive care unit (ICU) admission and death (severity score). Severity scores 0: asymptomatic patients who had no symptoms despite being tested positive for SARS-CoV-2 (ASYMP). Patients who were SARS-CoV-2 infected and developed symptoms (SYMP) were divided into four categories. Severity 1: patients who were screened at the hospital for COVID-19 but did not stay for regular admission. Severity 2: patients who were screened at the hospital for COVID-19 and went to non-ICU regular admission to treat their symptoms. Severity 3: patients who went to intensive ICU. Severity 4: patients who went to ICU with life support (i.e., mechanical ventilation at any point during their stay). Severity 5: patients who died from direct COVID-19 complications. The parameters displayed in the table (demographic features, HLA genotyping, clinical parameters, and prevalence of comorbidities) represent the number and percentages of patients within each disease severity. For the age parameter, median values are shown for each disease severity along with ranges (between brackets). The time between the onset of symptoms and the blood draw is shown as day-average numbers. The total number of comorbidities is the average of the sum of each patient’s comorbidities.

### Symptomatic and asymptomatic COVID-19 patient stratification based on disease severity

Following patient discharge, they were divided into six groups depending on the severity of their symptoms and their intensive care unit (ICU) and intubation (mechanical ventilation) status by medical practitioners. The scoring criteria were as follows: severity 5, patients who died from COVID-19 complications; severity 4, infected COVID-19 patients with severe disease who were admitted to the intensive care unit (ICU) and required ventilation support; severity 3, infected COVID-19 patients with severe disease that required enrollment in ICU, but without ventilation support; severity 2, infected COVID-19 patients with moderate symptoms that involved a regular hospital admission; severity 1, infected COVID-19 patients with mild symptoms; and severity 0, infected individuals with no symptoms. Among the 147 COVID-19 patients, subjects with a severity score of 0 were defined as asymptomatic, and subjects with a severity score of 1–5 were defined as symptomatic.

### Pre-pandemic healthy controls

Subsequently, we used 15 liquid-nitrogen frozen PBMCs samples (blood collected pre-COVID-19 in 2018) from HLA-A*02:01^+^/HLA-DRB1*01:01^+^ unexposed pre-pandemic healthy individuals (UPPHI, 8 men, 7 women; median age, 54 (20–76)] as controls to measure recalled SARS-CoV-2 cross-reactive T-cell responses. The class-II HLA status of each patient was first screened for HLA-DRB1*01:01 by PCR (**Supplementary Figure 1A**) (51). For class-I HLA, the screening was first performed (two-digit level) by HLA-A*02 flow cytometry staining (data not shown, mAbs clone BB7.2, BioLegend, San Diego, CA). The four-digit class-I HLA-A*02:01 subtype was subsequently screened by PCR (**Supplementary Figure 1B**) on blood samples (52).

### T-cell epitopes screening, selection, and peptide synthesis

CCCs/SARS-CoV-2 cross-reactive peptide epitopes from 12 SARS-CoV-2 proteins, including 27 9-mer long CD8^+^ T-cell epitopes (ORF1ab_84–92_, ORF1ab_1675–1683_, ORF1ab_2210–2218_, ORF1ab_2363–2371_, ORF1ab_3013–3021_, ORF1ab_3183–3191_, ORF1ab_3732–3740_, ORF1ab_4283–4291_, ORF1ab_5470–5478_, ORF1ab_6419–6427_, ORF1ab_6749–6757_, S_2–10_, S_691–699_, S_958–966_, S_976–984_, S_1000–1008_, S_1220–1228_, E_20–28_, E_26–34_, M_52–60_, M_89–97_, ORF6_3–11_, ORF7b_26–34_, ORF8a_31–39_, ORF8a_73–81_, ORF10_3–11_, and ORF10_5–13_) and 16 13-mer long CD4^+^ T-cell epitopes (ORF1a_1350–1365_, ORF1a_1801–1815_, ORF1ab_5019–5033_, ORF1ab_6088–6102_, ORF1ab_6420–6434_, S_1–13_, E_20–34_, E_26–40_, M_176–190_, ORF6_12–26_, ORF7a_1–15_, ORF7a_3–17_, ORF7a_98–112_, ORF7b_8–22_, ORF8b_1–15_, and N_388–403_) that we formerly identified were selected as we previously described (1) (**Table 2** and **Supplementary Table 1**). We used the Epitope Conservancy Analysis tool to compute the degree of identity of CD8^+^ and CD4^+^ T-cell epitopes within a given protein sequence of SARS-CoV-2 set at 100% identity level (1) (**Table 2** and **Supplementary Tables 1**, **2**). Peptides were synthesized (21st Century Biochemicals, Inc., Marlborough, MA), and the purity of peptides determined by both reversed-phase high-performance liquid chromatography and mass spectroscopy was over 95%.

**Table 2:** Percentages of identity and similarity scores (S^s^) between CCCs/SARS-CoV-2 cross-reactive CD4^+^ and CD8^+^ T cell epitopes.

	Peptide-epitope name/position	SARS-CoV-2 corresponding protein	SARS-CoV-2 peptide-epitope sequence	Correlation coefficient (R)*	Slope (S)*	Significance (i.e., *p*<0.05)? Y/N	Average IFNγ-SPOTs in HD (measure of observed T-cell cross-reactive response in HD individuals)
**CD4+ specific SARS-CoV-2 peptides** (class-II HLA-DRB1*01:01 restricted epitopes)	ORF1a_1350–1365_	Non-structural protein NSP3	KSAFYILPSIISNEK	−0.9418	−35.91	Y	52.5
	ORF1a_1801–1815_	Non-structural protein NSP3	ESPFVMMSAPPAQYE	−0.9480	−28.04	Y	20.5
	ORF1ab_5019–5033_	RdRP polymerase NSP12	PNMLRIMASLVLARK	−0.4115	−4.24	N	7.1
	ORF1ab_6088–6102_	Non-structural protein NSP14	RIKIVQMLSDTLKNL	−0.9581	−23.49	Y	10.6
	ORF1ab_6420–6434_	Non-structural protein NSP14	LDAYNMMISAGFSLW	−0.8711	−18.78	Y	3.1
	S_1–13_	Spike structural protein (Signal peptide)	MFVFLVLLPLVSS	−0.9262	−34.31	Y	39.1
	E_20–34_	Envelope structural protein	FLAFVVFLLVTLAIL	−0.8348	−18.83	Y	3.6
	E_26–40_	Envelope structural protein	FLLVTLAILTALRLC	−0.9172	−25.61	Y	12.6
	M_176–190_	Membrane structural protein	LSYYKLGASQRVAGD	−0.9421	−41.26	Y	40.7
	ORF6_12–26_	ORF6 accessory protein	AEILLIIMRTFKVSI	−0.9378	−31.67	Y	41.0
	ORF7a_1–15_	ORF7a accessory protein	MKIILFLALITLATC	−0.9390	−17.96	Y	5.1
	ORF7a_3–17_	ORF7a accessory protein	IILFLALITLATCEL	−0.8915	−17.76	Y	2.8
	ORF7a_98–112_	ORF7a accessory protein	SPIFLIVAAIVFITL	−0.6833	−9.343	N	1.8
	ORF7b_8–22_	ORF7b accessory protein	DFYLCFLAFLLFLVL	−0.8715	−21.66	Y	3.2
	ORF8b_1–15_	ORF8 accessory protein	MKFLVFLGIITTVAA	−0.9191	−29.04	Y	32.4
	N_388–403_	Nucleocapsid structural protein	KQQTVTLLPAADLDDF	−0.8905	−32.13	Y	23.4
**CD8+ specific SARS-CoV-2 peptides** (class-I HLA-A*02:01 restricted epitopes)	ORF1ab_84–92_	Non-structural protein NSP1	VMVELVAEL	−0.8984	−20.28	Y	1.5
	ORF1ab_1675–1683_	Non-structural protein NSP3	YLATALLTL	−0.9469	−38.91	Y	54.6
	ORF1ab_2210–2218_	Non-structural protein NSP3	CLEASFNYL	–0.7327	–9.93	N	0.7
	ORF1ab_2363–2371_	Non-structural protein NSP3	WLMWLIINL	–0.8962	–14.36	Y	9.9
	ORF1ab_3013–3021_	Non-structural protein NSP4	SLPGVFCGV	–0.5539	–3.89	N	20.4
	ORF1ab_3183–3191_	Non-structural protein NSP4	FLLNKEMYL	–0.8314	–18.60	Y	15.5
	ORF1ab_3732–3740_	Non-structural protein NSP6	SMWALIISV	–0.8909	–19.89	Y	41.0
	ORF1ab_4283–4291_	Non-structural protein NSP10	YLASGGQPI	–0.9269	–30.27	Y	50.2
	ORF1ab_5470–5478_	Non-structural protein NSP13	KLSYGIATV	–0.4496	–7.852	N	55.2
	ORF1ab_6419–6427_	Non-structural protein NSP14	YLDAYNMMI	–0.9026	–25.27	Y	45.7
	ORF1ab_6749–6757_	Non-structural protein NSP15	LLLDDFVEI	–0.9460	–35.39	Y	55.5
	S_2–10_	Spike structural protein (Signal peptide)	FVFLVLLPL	–0.9541	–32.27	Y	43.9
	S_691–699_	Spike structural protein (S1/S2 cleavage)	SIIAYTMSL	–0.7151	–13.90	N	17.7
	S_958–966_	Spike structural protein (S2: between HR1 and HR2)	ALNTLVKQL	–0.9425	–34.44	Y	40.2
	S_976–984_	Spike structural protein (S2: between HR1 and HR2)	VLNDILSRL	–0.6020	–24.77	N	62.8
	S_1000–1008_	Spike structural protein (S2: between HR1 and HR2)	RLQSLQTYV	–0.9408	–34.98	Y	51.0
	S_1220–1228_	Spike structural protein (CT: cytoplasmic domain)	FIAGLIAIV	–0.9488	–52.81	Y	72.1
	E_20–28_	Envelope structural protein	FLAFVVFLL	–0.8656	–17.72	Y	21.5
	E_26–34_	Envelope structural protein	FLLVTLAIL	–0.9408	–31.78	Y	32.1
	M_52–60_	Membrane structural protein	IFLWLLWPV	–0.9083	–27.55	Y	31.1
	M_89–97_	Membrane structural protein	GLMWLSYFI	–0.9141	–22.70	Y	23.6
	ORF6_3–11_	ORF6 accessory protein	HLVDFQVTI	–0.8881	–18.77	Y	21.9
	ORF7b_26–34_	ORF7b accessory protein	IIFWFSLEL	–0.8960	–18.32	Y	11.9
	ORF8a_31–39_	ORF8 accessory protein	YVVDDPCPI	–0.8756	–16.70	Y	18.6
	ORF8a_73–81_	ORF8 accessory protein	YIDIGNYTV	–0.8775	–15.67	Y	17.1
	ORF10_3–11_	ORF10 accessory protein	YINVFAFPF	–0.9539	–38.33	Y	44.6
	ORF10_5–13_	ORF10 accessory protein	NVFAFPFTI	–0.9477	–25.64	Y	41.5

*To assess (for each individual SARS-CoV-2 epitope) the magnitude of the correlation between the breadth of this epitope-specific T-cell response and the protection against severe COVID-19
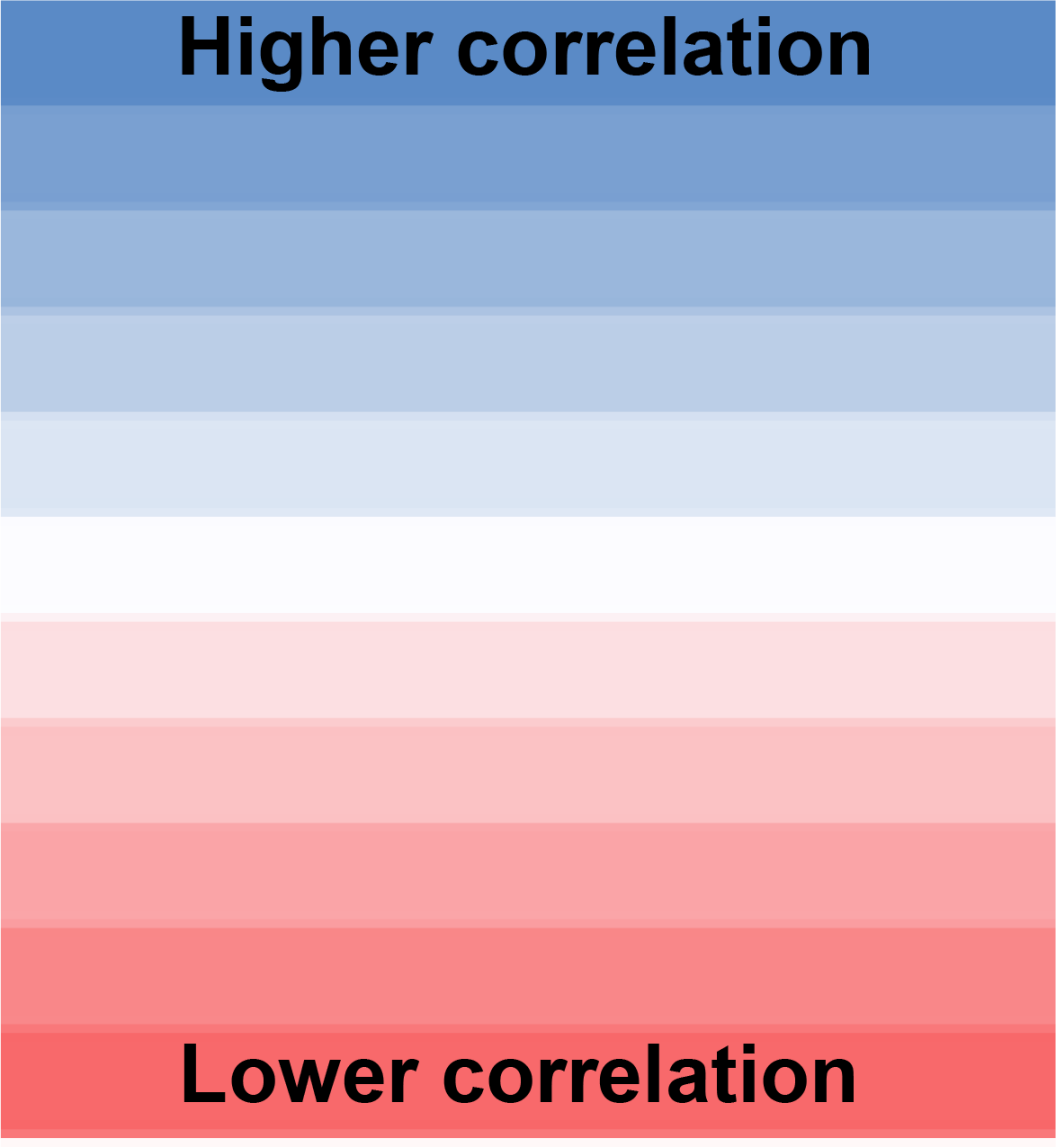

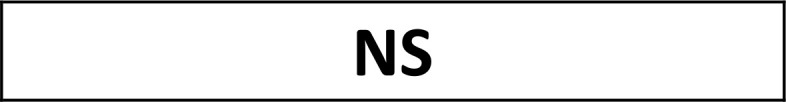

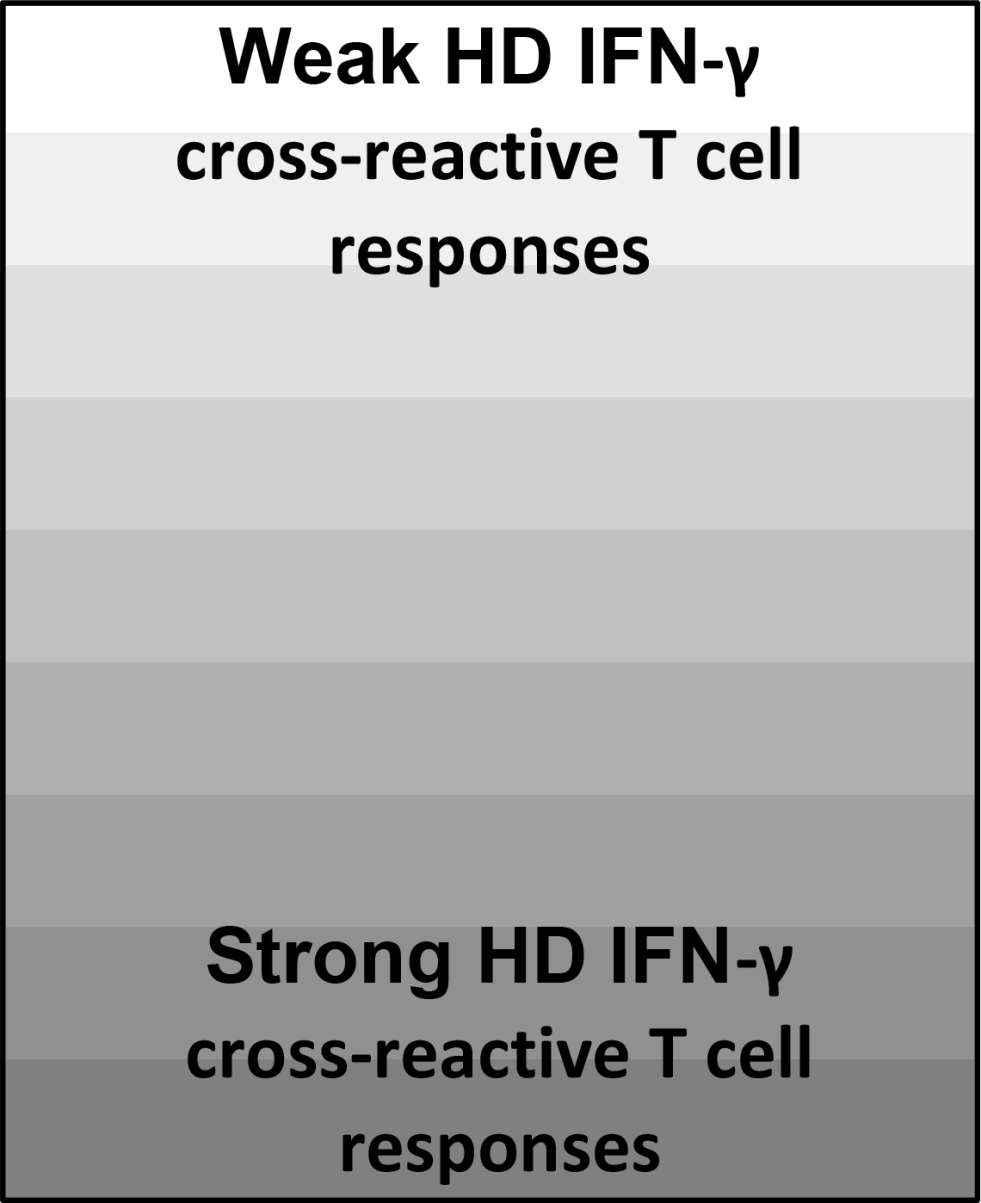

Matching CCCs peptides were chosen after combining both MSA and ECT analysis (see Materials and methods). Each panel represents the alignment of epitopes from SARS-CoV-2 and the four main and seasonal α and β species of common cold coronavirus (CCCs) (i.e., α-CCC-NL63, α-CCC-229E, β-CCC-HKU1, and β-CCC-OC43). The SARS-CoV-2 peptide sequence is set as 100% identity. The amino acids color code was generated with Gecos software (https://gecos.biotite-python.org) using the following parameters: geckos –matrix BLOSUM62 –min 60 –max 75 –f. The distance between two amino acids in the substitution matrix (BLOSUM62) corresponds to the perceptual visual differences in the color scheme. Similarity scores (S^S^) based on such matrix are a good predictive measure of potential cross-reactivity (along with % of peptide identity). S^S^ ≥ 0.80 and %id ≥ 67% are in red. Identity percentages, similarity scores, conservation, and consensus sequences are indicated in each panel. For each SARS-CoV-2 epitope, the significance (p < 0.05) of each correlation is also indicated, along with the magnitude of the T-cell cross-reactive response measured by IFN-γ ELISpots in HD individuals.

### Blood differential test

Total white blood cell (WBC) count and lymphocyte count per microliter of blood were performed by the clinicians at the University of California Irvine Medical Center, using a CellaVision™ DM96 automated microscope. Monolayer smears were prepared from anticoagulated blood and stained using the May Grunwald Giemsa (MGG) technique. Subsequently, slides were loaded onto the DM96 magazines and scanned using a ×10 objective focused on nucleated cells to record their exact position. Images were obtained using the ×100 oil objective and analyzed by artificial neural network (ANN).

### Peripheral blood mononuclear cells isolation and T-cell stimulation

Peripheral blood mononuclear cells (PBMCs) from COVID-19 patients were isolated from the blood using Ficoll (GE Healthcare) density gradient media and transferred into 96-well plates at a concentration of 2.5 × 10^6^ viable cells per ml in 200 µl (0.5 × 10^6^ cells per well) of RPMI-1640 media (Hyclone) supplemented with 10% (v/v) FBS (HyClone), sodium pyruvate (Lonza), L-glutamine, nonessential amino acids, and antibiotics (Corning). A fraction of the blood was kept separated to perform HLA genotyping of the patients and select only the HLA-A*02:01 and/or DRB1*01:01 positive individuals (**Supplementary Figure 1**). Fresh peripheral blood mononuclear cells (PBMCs) were used in this study, as they generally have higher viability and functionality compared to frozen PBMCs. Freezing and thawing can lead to cell damage and loss of T-cell functionality, which may affect the accuracy and reliability of experimental results. Frozen PBMCs may exhibit altered activation status compared to fresh cells. Cryopreservation can induce stress responses in cells, leading to changes in their activation state and potentially affecting immune response assays. In the context of COVID-19 research, where precise characterization of immune responses is crucial for understanding disease pathogenesis, vaccine development, and treatment strategies, using fresh PBMCs ensures the accuracy and reliability of experimental results. A side-by-side comparison of frozen and fresh PBMCs and pre-pandemic healthy control PBMCs yielded no significant difference. PBMCs were stimulated with 10 µg/ml of each one of the 43 individual CCCs/SARS-CoV-2 cross-reactive peptide epitopes (27 CD8^+^ T-cell peptides and 16 CD4^+^ T-cell peptides) and incubated in a humidified chamber with 5% CO_2_ at 37°C (**Supplementary Figure 2A**). Post-incubation, cells were stained by flow cytometry analysis or transferred onto IFN-γ ELISpot plates. The same isolation protocol was followed for HD samples obtained in 2018. Ficoll was kept frozen in liquid nitrogen in FBS DMSO 10%; after thawing, HD PBMCs were stimulated similarly for the IFN-γ ELISpot technique.

### ELISpot assay

COVID-19 patients were first screened for their HLA status (DRB1*01:01^+^ positive = 92 out of 600 tested, HLA-A*02:01^+^ positive = 71, DRB1*01:01^+^ and HLA-A*02:01^+^ positive = 16) (**Supplementary Figure 1**). The 92 DRB1*01:01 positive individuals were then used to assess the CD4^+^ T-cell response against CCCs/SARS-CoV-2 cross-reactive class-II restricted epitopes by IFN-γ ELISpot (**Supplementary Figure 2**). Similarly, we assessed the CD8^+^ T-cell response against our CCCs/SARS-CoV-2 cross-reactive class-I restricted epitopes in the 71 HLA-A*02:01 positive individuals representing different disease severity categories (**Table 1**).

All ELISpot reagents were filtered through a 0.22-µm filter. Wells of 96-well Multiscreen HTS Plates (Millipore, Billerica, MA) were pre-wet with 30% ethanol for 60 s and then coated with 100 µl primary anti-IFN-γ antibody solution (10 µg/ml of 1-D1K coating antibody from Mabtech, Cincinnati, OH) OVN at 4°C. After washing, the plate was blocked with 200 µl of RPMI media plus 10% (v/v) FBS for 2 h at room temperature to prevent nonspecific binding. After 24 h, following the blockade, the peptide-stimulated cells from the patient’s PBMCs (0.5 × 10^6^ cells/well) were transferred into the ELISpot-coated plates. PHA-stimulated or non-stimulated cells (DMSO) were used as positive or negative controls of T-cell activation, respectively. Upon incubation in a humidified chamber with 5% CO_2_ at 37°C for an additional 48 h, cells were next washed using PBS and PBS-Tween 0.02% solution. Next, 100 µl of biotinylated secondary anti-IFN-γ antibody (1 µg/ml, clone 7-B6-1, Mabtech) in blocking buffer (PBS 0.5% FBS) was added to each well. Following a 2-h incubation followed by washing, wells were incubated with 100 µl of HRP-conjugated streptavidin (1:1,000) for 1 h at room temperature. Lastly, wells were incubated for 15–30 min with 100 µl of TMB detection reagent at room temperature, and spots were counted both manually and by an automated ELISpot reader counter (ImmunoSpot Reader, Cellular Technology, Shaker Heights, OH).

### Flow cytometry analysis

Surface markers detection and flow cytometry analysis were performed on 147 patients after 72 h of stimulation with each CCCs/SARS-CoV-2 cross-reactive class-I or class-II restricted peptide; PBMCs (0.5 × 10^6^ cells) were stained (**Supplementary Figure 2**). First, the cells were stained with a live/dead fixable dye (Zombie Red dye, 1/800 dilution—BioLegend, San Diego, CA) for 20 min at room temperature, to exclude dying/apoptotic cells. Subsequently, cells were stained for 45 min at room temperature with five different HLA-A*02*01 restricted tetramers and/or five HLA-DRB1*01:01 restricted tetramers (PE labeled) specific toward the CCCs/SARS-CoV-2 cross-reactive CD8^+^ T-cell epitopes Orf1ab_2210–2218_, Orf1ab_4283–4291_, S_976–984_, S_1220–1228_, and ORF10_3–11_ and toward the CCCs/SARS-CoV-2 cross-reactive CD4^+^ T-cell epitopes ORF1a_1350–1365_, S_1–13_, E_26–40_, M_176–190_, and ORF6_12–26_, respectively. Cells were alternatively stained with the EBV BMLF-1_280–288_-specific tetramer for controls (53) (**Supplementary Figure 3**). We optimized our tetramer staining according to protocol instructions published by Dolton et al. (54). We stained HLA-A*02*01- HLA-DRB1*01:01-negative patients with our 10 tetramers as a negative control aiming to assess tetramers staining specificity. Subsequently, we used anti-human antibodies for surface-marker staining: anti-CD45 (BV785, clone HI30—BioLegend), anti-CD3 (Alexa700, clone OKT3—BioLegend), anti-CD4 (BUV395, clone SK3—BD), anti-CD8 (BV510, clone SK1—BioLegend), anti-TIGIT (PercP-Cy5.5, clone A15153G—BioLegend), anti-TIM-3 (BV 711, clone F38-2E2—BioLegend), anti-PD1 (PE-Cy7, clone EH12.1—BD), anti-CTLA-4 (APC, clone BNI3—BioLegend), anti-CD137 (APC-Cy-7, clone 4B4-1—BioLegend), and anti-CD134 (BV650, clone ACT35—BD). mAbs against these various cell markers were added to the cells, either *ex vivo* or *in vitro*, in phosphate-buffered saline (PBS) containing 1% FBS and 0.1% sodium azide [fluorescence-activated cell sorter (FACS) buffer] and incubated for 30 min at 4°C. Cells were then washed twice with FACS buffer and fixed with paraformaldehyde 4% (PFA, Affymetrix, Santa Clara, CA). A total of ∼200,000 lymphocyte-gated PBMCs (140,000 alive CD45^+^) were acquired by Fortessa X20 (Becton Dickinson, Mountain View, CA) and analyzed using FlowJo software (TreeStar, Ashland, OR). The gating strategy is detailed in **Supplementary Figure 2B**.

### TaqMan quantitative polymerase reaction assay for the detection of CCC species in UPPHI and in COVID-19 patients

To detect common cold coronavirus co-infection in COVID-19 patients, Taqman PCR assays were performed on a total of 85 patients distributed into each different category of disease severity (9 ASYMP, 6 patients of category 1, 32 patients of category 2, 9 patients of category 3, 15 patients of category 4, and 14 patients of category 5). Nucleic acid was first extracted from each nasopharyngeal swab sample using Purelink Viral RNA/DNA mini kit (Thermo Fisher Scientific, Waltham, MA) according to the manufacturer’s instructions. Subsequently, extracted RNA samples were quantified using Qubit and BioAnalyzer. cDNA was synthesized from 10 μL of RNA eluate using random hexamer primers and SuperScript II Reverse Transcriptase (Applied Biosystems, Waltham, MA). The subsequent RT-PCR screening of the enrolled subjects for the four CCCs was performed using specific sets of primers and probes (55).

CCC-229E, CCC-OC43, and CCC-NL63 RT-PCR assays were performed using the following conditions: 50°C for 15 min followed by denaturation at 95°C for 2 min, 40 cycles of PCR performed at 95°C for 8 s, extending and collecting a fluorescence signal at 60°C for 34 s (56). For CCC-HKU1, the amplification conditions were 48°C for 15 min, followed by 40 cycles of 94°C for 15 s and 60°C for 15 s. For each virus, when the Ct-value generated was <35, the specimen was considered positive. When the Ct-value was relatively high (35 ≤ Ct < 40), the specimen was retested twice and considered positive if the Ct-value of any retest was <35 (57).

### Identity and similarity analysis of CCCs/SARS-CoV-2 cross-reactive epitopes

To assess the % identity (%id) of CCCs/SARS-CoV-2 cross-reactive CD4^+^ and CD8^+^ T-cell peptide epitopes, we first identified the best matching CCCs peptide across the CCCs proteomes (**Table 2**). The full CCCs proteomes sequences were obtained from the National Center for Biotechnology Information (NCBI) GenBank [MH940245.1 (CCC-HUK1), MN306053.1 (CCC-OC43), KX179500.1 (CCC-NL63), and MN306046.1 (CCC-229E)]. We processed this in the following three steps. (1) Corresponding CCCs peptides were determined after protein sequence alignments of all four homologous CCCs proteins plus the SARS-CoV-2 related one using various multiple sequences alignments (MSA) algorithms ran in JALVIEW, MEGA11, and M-coffee software’s (i.e., ClustalO, Kalign3, and M-coffee—the latter computing alignments by combining a collection of multiple alignments from a library constituted with the following algorithms: T-Coffee, PCMA, MAFFT, ClustalW, Dialigntx, POA, MUSCLE, and Probcons). Furthermore, we confirmed our results with global and local pairwise alignments (Needle and Water algorithms ran in Biopython) performed to confirm the results. In case of obtaining different results with the various algorithms, the epitope sequence with the highest BLOSUM62-sum score compared to the SARS-CoV-2 epitope set as reference was selected (**Table 2** and **Supplementary Tables 1**, **2**). We calculated the % of identity and similarity score S^s^ with its related SARS-CoV-2 epitope, for each of these CCCs peptides (**Supplementary Tables 1-3**). The peptide similarity score S^s^ calculation is based on the method reported by Sune Frankild et al. (58) and the BLOSUM62 matrix to calculate a BLOSUM62 sum (using the Bio.SubsMat.MatrixInfo package in Biopython) between a pair of peptides (peptide “x” from SARS-CoV-2 and “y” from one CCC) and compared their similarity. 0 ≤ S^s^ ≤ 1: the closest S^s^ is to 1, the highest is the potential for T-cell cross-reactivity response toward the related pair of peptides (58). We used a threshold of S^s^≥0.8 to discriminate between highly similar and non-similar peptides. (2) Then, we examined if other parts of each of the CCCs proteome (without restricting our search only to peptides present in CCCs homologous proteins) could contain better matching peptides than the CCCs peptides reported in **Supplementary Tables 1**-**3**. First, for each one of our 16 CD4^+^ and 27 CD8^+^ SARS-CoV-2 epitopes, we spanned the entire proteome of each CCCs using the Epitope Conservancy Tool (ECT: http://tools.iedb.org/conservancy/—with a conservancy threshold of 20%). All the CCCs peptides from the top query (i.e., with the highest % of identity) were reported for every four CCCs in **Supplementary Tables 1**-**3**. Second, among these returned top queries (peptides with the same highest % identity), we picked the one with the highest similarity score S^s^ (bolded in **Supplementary Tables 1**-**3**—right column). (3) We compared this peptide with the one previously found in **Supplementary Table 1** based on MSA. When both methods returned the same peptide (from the same protein), we kept it (peptides highlighted in beige in **Supplementary Tables 1**-**3**). When both matching peptides (using the two different methods) were found to be different, we compared (i) %id_MSA_ with %id_ECT_ and (ii) S^s^_MSA_ with S^s^_ECT_. If %id_MSA_ ≤ %id_ECT_ but S^s^_MSA_ ≥ S^s^_ECT_, we kept the CCCs peptide found following the MSA method; however, if %id_MSA_ ≤ %id_ECT_ and S^s^_MSA_ < S^s^_ECT_, we then picked the CCC peptide found using the ECT instead of the one found using MSA (peptides not highlighted in **Supplementary Tables 1**-**3**).

Using the %id and the calculated similarity score with the SARS-CoV-2 epitopes, all related CCCs’ best-matching peptides are reported in **Supplementary Tables 1**-**3**. They were then evaluated based on their potential to induce a cross-reactive T-cell response (**Supplementary Tables 1**-**3**): (0), CCC best matching peptide with low to no potential to induce a cross-reactive response toward the corresponding SARS-CoV-2 epitope and *vice versa* (%id with the corresponding SARS-CoV-2 epitope < 67% and similarity score S^s^ < 0.8); (0.5), CCC best matching peptide that may induce a cross-reactive response (%id with the corresponding SARS-CoV-2 epitope ≥ 67% OR similarity score S^s^ ≥ 0.8); and (1), CCC best-matching peptide is very likely to induce a cross-reactive response (%id ≥ 67% and S^s^ ≥ 0.8).

### Identification of potential cross-reactive peptides in non-CCC human pathogens and vaccines

We took advantage of the database generated by Pedro A. Reche (59). Queries to find matching peptides with our SARS-CoV-2-derived CD4^+^ and CD8^+^ epitopes were performed from the data gathered; only peptides sharing a %id ≥ 67% with our corresponding SARS-CoV-2 epitope were selected. The corresponding similarity score S^s^ was calculated.

### Statistical analyses

To assess the linear negative relationship between COVID-19 severity and the magnitude of each SARS-CoV-2 epitope-specific T-cell response, correlation analysis using GraphPad Prism version 8 (La Jolla, CA) was performed to calculate Pearson correlation coefficients (R), coefficient of determination (R^2^), and associated *p*-value (correlation statistically significant for *p* ≤ 0.05). The slope (S) of the best-fitted line (dotted line) was calculated in Prism by linear regression analysis. The same statistical analysis was performed to compare the cross-reactive pre-existing T-cell response in unexposed pre-pandemic healthy individuals (UPPHI) with the slope S (magnitude of the correlation between this epitope-specific T-cell response in SARS-CoV-2-infected patients and the protection against severe COVID-19). Absolute WBCs and lymphocyte cell numbers (per µL of blood, measured through BDT), corresponding lymphocytes percentages/ratio, flow cytometry data measuring CD3^+^/CD8^+^/CD4^+^ cell percentages and the percentages detailing the magnitude (Tetramer^+^ T cell %), and the quality (% of PD1^+^/TIGIT^+^, CTLA-4^+^/TIM3^+^ or AIMs^+^ cells) of the CD4^+^ and CD8^+^ SARS-CoV-2-specific T cells, were compared across groups and categories of disease severity by one-way ANOVA multiple tests. ELISpot SFCs data were compared by Student’s *t*-tests. Data are expressed as the mean ± SD. Results were considered statistically significant at *p* ≤ 0.05. To evaluate whether the differences in frequencies of RT-PCR positivity to the four CCCs across categories of disease severity were significant, we used the Chi-squared test or Fisher’s exact test.

## Results

### Higher magnitudes of common cold coronavirus/SARS-CoV-2 cross-reactive CD4^+^ T-cell responses detected in unvaccinated asymptomatic COVID-19 patients

**Figure 1:**
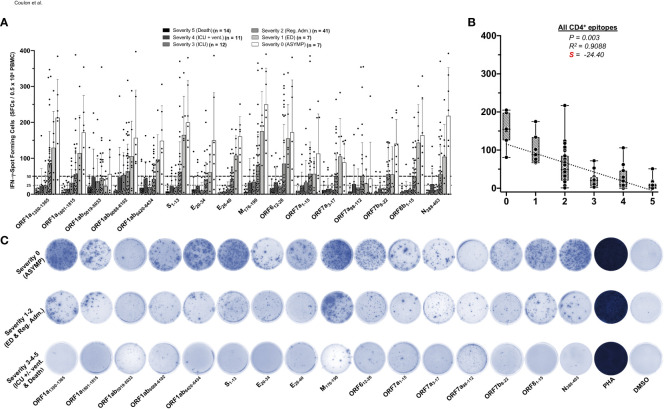
IFN-γ-producing CD4^+^ T-cell responses to CCCs/SARS-CoV-2 cross-reactive epitopes in unvaccinated COVID-19 patients with various degrees of disease severity. PBMCs from HLA-DRB1*01:01-positive COVID-19 patients (*n* = 92 are HLA-DRB1*01:01-positive out of 600 tested) were isolated and stimulated for a total of 72 h with 10 µg/ml of each of the previously identified 16 CCCs/SARS-CoV-2 cross-reactive CD4^+^ T cell epitope peptides. The number of IFN-γ-producing CD4^+^ T cells was quantified in each of the 92 patients using ELISpot assay. **(A)** Average/mean numbers (± SD) of IFN-γ-spot forming cells (SFCs) after CD4^+^ T-cell peptide-stimulation detected in each of the 92 COVID-19 patients divided into six groups based on disease severity scored 0–5, as described in Materials and methods, and as identified by six columns on a grayscale (black columns = severity 5, to white columns = severity 0) is shown. Dotted lines represent an arbitrary threshold set as a cutoff of the positive response. A mean SFC between 25 and 50 SFCs corresponds to a medium/intermediate response, whereas a strong response is defined for mean SFCs > 50 per 0.5 × 10^6^ stimulated PBMCs. **(B)** Correlation between the overall number of IFN-γ-producing CD4^+^ T cells induced by each of the 16 CCCs/SARS-CoV-2 cross-reactive CD4^+^ T-cell epitope peptides in each of the six groups of COVID-19 patients with various disease severity. The coefficient of determination (R^2^) is calculated from the Pearson correlation coefficients (R). The associated *p*-value and the slope (S) of the best-fitted line (dotted line) calculated by linear regression analysis are indicated. The gray-hatched boxes in the correlation graphs extend from the 25th to 75th percentiles (hinges of the plots) with the median represented as a horizontal line in each box and the extremity of the vertical bars showing the minimum and maximum values. **(C)** Representative spots images of the IFN-γ-spot forming cells (SFCs) induced by each of the 16 CCCs/SARS-CoV-2 cross-reactive CD4^+^ T cell epitope peptides in three representative patients, each falling into one of three groups of disease category: the unvaccinated asymptomatic COVID-19 patients (ASYMP, severity score 0), unvaccinated COVID-19 patients who developed mild to moderate disease (severity scores 1 and 2) and unvaccinated severely ill COVID-19 patients and unvaccinated patients with fatal COVID-19 outcomes (severity scores 3–5). PHA was used as a positive control of T-cell activation. Unstimulated negative control SFCs (DMSO—no peptide stimulation) were subtracted from the SFC counts of peptides-stimulated cells. Results are representative of two independent experiments and were considered statistically significant at *p* ≤ 0.05.

We first compared SARS-CoV-2-specific CD4^+^ T-cell responses in unvaccinated asymptomatic COVID-19 patients (those individuals who never develop any COVID-19 symptoms despite being infected with SARS-CoV-2) to unvaccinated symptomatic (those patients who developed severe to fatal COVID-19 symptoms) (**Figure 1**). We used 16 recently identified HLA-DR-restricted CD4^+^ T-cell epitopes that are highly conserved between human SARS-CoVs and CCCs (1). We enrolled 92 unvaccinated HLA-DRB1*01:01^+^ COVID-19 patients, who were genotyped using PCR (**Supplementary Figure 1**) and divided into six groups, based on the level of severity of their COVID-19 symptoms (from severity 5 to severity 0, assessed at discharge). Clinical and demographic characteristics of this cohort of COVID-19 patients are detailed in **Table 1**. Fresh PBMCs were isolated from these COVID-19 patients, on average within 4.8 days after reporting a first COVID-19 symptom or a first PCR-positive test (**Table 1**). PBMCs were then stimulated *in vitro* for 72 h using each of the 16 CD4^+^ T-cell peptide epitopes, as detailed in *Materials and methods* and illustrated in **Supplementary Figure 2**. The frequency of responding IFN-γ-producing CD4^+^ T cells specific to individual epitopes was quantified, in each of the six groups of COVID-19 patients, using ELISpot assay (i.e., number of IFN-γ-spot forming CD4^+^ T cells or “SFCs”) (**Figure 1**). A positive IFN-γ-producing CD4^+^ T-cell responses was determined as the mean SFCs > 50 per 0.5 × 10^6^ PBMCs fixed as threshold.

Overall, the highest frequencies of CCCs/SARS-CoV-2 cross-reactive epitope-specific IFN-γ-producing CD4^+^ T cells were detected in the unvaccinated COVID-19 patients with less severe disease (i.e., severity 0, 1, and 2, **Figures 1A, B**). In contrast, the lowest frequencies of CCCs/SARS-CoV-2 cross-reactive IFN-γ-producing CD4^+^ T cells were detected in unvaccinated severely ill COVID-19 patients (severity scores 3 and 4) and in unvaccinated COVID-19 patients with fatal outcomes (severity score of 5, **Figures 1A, B**).

Pearson correlation analysis was performed to determine the linear correlation between the magnitude of CD4^+^ T-cell responses directed toward each of the 16 highly conserved SARS-CoV-2 epitopes and the severity of COVID-19 symptoms. A negative correlation is usually considered strong when the coefficient R-value is between −0.7 and −1. Except for the ORF1ab_5019–5033_ and ORF7a_98–112_ epitopes, we found that a strong positive linear correlation existed between the high magnitude of IFN-γ-producing CD4^+^ T-cell responses specific to 14 CD4^+^ T-cell epitopes and the “natural protection” observed in unvaccinated asymptomatic COVID-19 patients (**Figures 1A, C**). This positive correlation existed regardless of whether CD4^+^ T cells cross-recognized structural, non-structural, or accessory SARS-CoV-2 antigens. Cross-reactive IFN-γ-producing CD4^+^ T-cell responses, specific to M_176–190_, ORF1a_1350–1365_, S_1–13_, N_388–403_, and ORF6_12–26_, and to a slightly lesser extent to ORF8b_1–15_ and ORF1a_1801–1815_, were associated with a low COVID-19 severity score (i.e., negatively correlated with an R close to −1) and a very strong negative slope (−41.26 < S < −28.04). Comparatively, the CD4^+^ T-cell responses against E_26–40_, ORF1ab_6088–6102_, ORF7b_8–22_, E_20–34_, ORF1ab_6420–6434_, ORF7a_1–15_, and ORF7a_3–17_ were also negatively associated with severe disease in patients, but to a lesser degree (relatively less negative slope: −25.61 < S < −17.76) (**Figure 1A** and **Supplementary Figure 4**). In contrast, no significant correlation was found between the magnitude of IFN-γ-producing CD4^+^ T-cell responses directed toward ORF1ab_5019–5033_ and ORF7a_98–112_ epitopes and the disease severity (*p* > 0.05). For the ORF1ab_5019–5033_ and ORF7a_98–112_ epitopes, the slope was comparatively weak: only slightly negative with S > −10 (**Figure 1A** and **Supplementary Figure 4**).

Taken together, these results (i) demonstrate an overall higher magnitude of CCCs/SARS-CoV-2 cross-reactive CD4^+^ T-cell responses present in unvaccinated asymptomatic COVID-19 patients. In contrast, a lower magnitude of CCCs/SARS-CoV-2 cross-reactive CD4^+^ T-cell responses were detected in unvaccinated severely ill COVID-19 patients and to patients with fatal COVID-19 outcomes and (ii) suggest a crucial role of CCCs/SARS-CoV-2 cross-reactive CD4^+^ T cells, directed towards structural, non-structural, and accessory protein antigens, in protection from symptomatic and fatal Infections in unvaccinated COVID-19 patients.

### Higher magnitudes of CD8^+^ T-cell responses to common cold coronavirus/SARS-CoV-2 cross-reactive epitopes detected in unvaccinated asymptomatic COVID-19 patients

**Figure 2:**
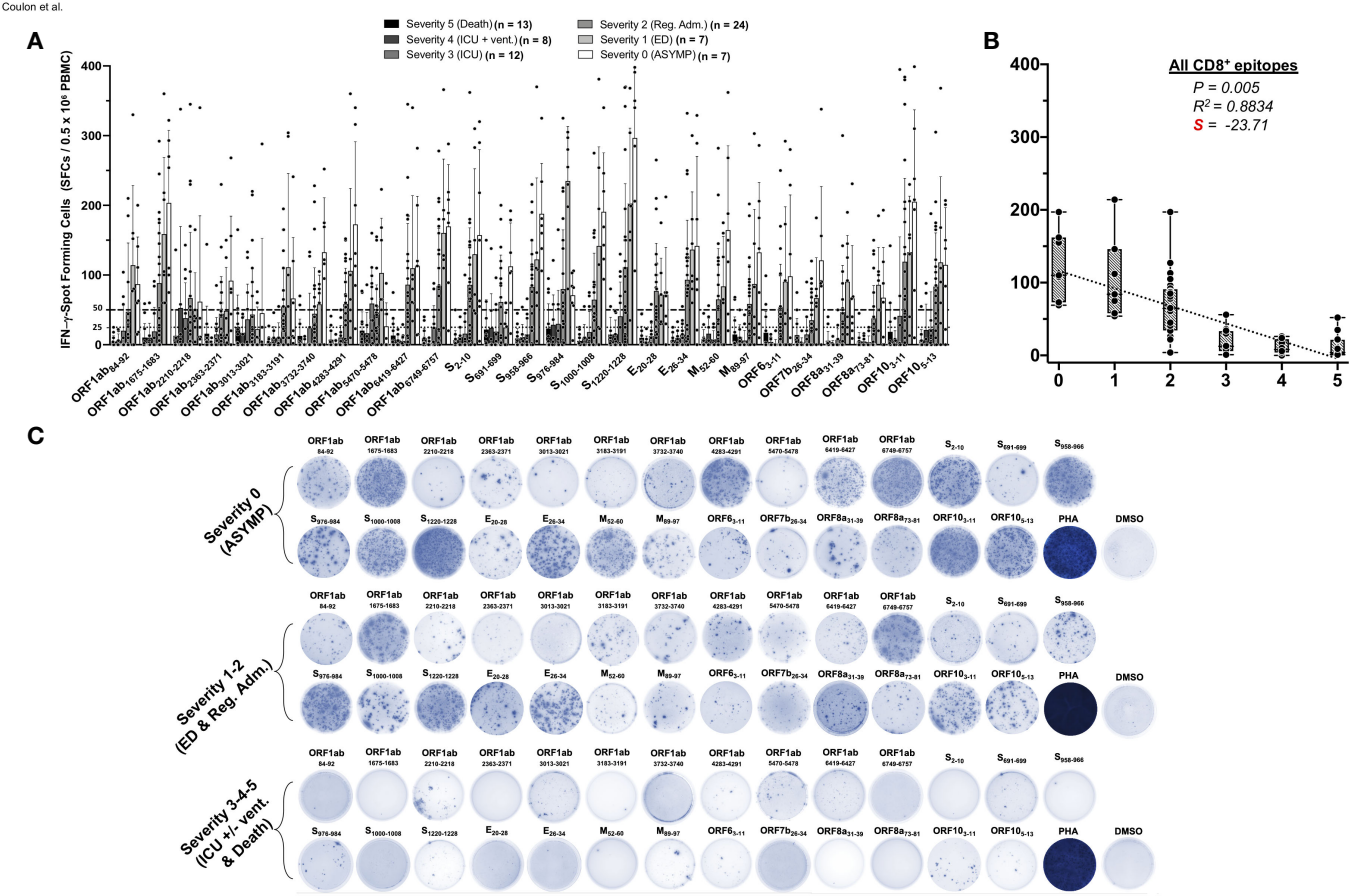
IFN-γ-producing CD8^+^ T-cell responses to CCCs/SARS-CoV-2 cross-reactive epitopes in unvaccinated COVID-19 patients with various degrees of disease severity. PBMCs from HLA-A*02:01-positive COVID-19 patients (*n* = 71) were isolated and stimulated for a total of 72 h with 10 µg/ml of each of the previously identified 27 CCCs/SARS-CoV-2 cross-reactive CD8^+^ T-cell epitope peptides. The number of IFN-γ-producing CD8^+^ T cells was quantified in each of the 71 patients using ELISpot assay. Panel **(A)** shows the average/mean numbers (± SD) of IFN-γ-spot forming cells (SFCs) after CD8^+^ T-cell peptide stimulation detected in each of the 71 COVID-19 patients divided into six groups based on disease severity scored 0–5, as described in *Materials and methods*, and as identified by six columns on a grayscale (Black columns = severity 5, to white columns = severity 0). Dotted lines represent an arbitrary threshold set as a cutoff of the positive response. A mean SFCs between 25 and 50 SFCs corresponds to a medium/intermediate response, whereas a strong response is defined for mean SFCs > 50 per 0.5 × 10^6^ stimulated PBMCs. **(B)** Correlation between the overall number of IFN-γ-producing CD8^+^ T cells induced by each of the 27 CCCs/SARS-CoV-2 cross-reactive CD8^+^ T-cell epitope peptides in each of the six groups of COVID-19 patients with various disease severity. The coefficient of determination (R^2^) is calculated from the Pearson correlation coefficients (R). The associated *p*-value and the slope (S) of the best-fitted line (dotted line) calculated by linear regression analysis are indicated. The gray-hatched boxes in the correlation graphs extend from the 25th to 75th percentiles (hinges of the plots) with the median represented as a horizontal line in each box and the extremity of the vertical bars showing the minimum and maximum values. **(C)** Representative spots images of the IFN-γ-spot forming cells (SFCs) induced by each of the 27 CCCs/SARS-CoV-2 cross-reactive CD8^+^ epitope peptides in three representative patients, each falling into one of three groups of disease category: the unvaccinated asymptomatic COVID-19 patients (ASYMP, severity score 0), unvaccinated COVID-19 patients who developed mild to moderate disease (severity scores 1 and 2), and unvaccinated severely ill COVID-19 patients and unvaccinated patients with fatal COVID-19 outcomes, (severity scores 3–5). PHA was used as a positive control of T-cell activation. Unstimulated negative control SFCs (DMSO—no peptide stimulation) were subtracted from the SFC counts of peptides-stimulated cells. Results are representative of two independent experiments and were considered statistically significant at *p* ≤ 0.05.

We next compared the CCCs/SARS-CoV-2 cross-reactive CD8^+^ T-cell responses in unvaccinated asymptomatic individuals vs. unvaccinated symptomatic COVID-19 patients (**Figure 2**). We used 27 recently identified HLA-A*0201-restricted CD8^+^ T-cell epitopes that are highly conserved between human SARS-CoVs and CCCs (1). We enrolled 71 unvaccinated HLA-A*0201^+^ COVID-19 patients, who were genotyped using PCR (**Supplementary Figure 1**) and divided into six groups based on the severity of COVID-19 symptoms (i.e., severity 5 to severity 0, **Table 1**). Fresh PBMCs were isolated from COVID-19 patients on an average of 4.8 days after reporting initial COVID-19 symptoms or a first PCR-positive test (in the case of asymptomatic). Subsequently, PBMCs were stimulated *in vitro* for 72 h using each of the 27 HLA-A*0201-restricted CD8^+^ T-cell peptide epitopes (**Supplementary Figure 2**). The frequency of responding IFN-γ-producing CD8^+^ T cells specific to individual epitopes was quantified, in each of the six groups of COVID-19 patients, using the ELISpot assay (i.e., number of IFN-γ-spot forming CD8^+^ T cells or “SFCs”) (**Figure 2**).

Overall, the highest frequencies of CCCs/SARS-CoV-2 cross-reactive epitope-specific functional IFN-γ-producing CD8^+^ T cells (mean SFCs > 50 per 0.5 × 10^6^ PBMCs) were detected in the three groups of unvaccinated COVID-19 patients who presented little to no severe COVID-19 symptoms (i.e., severity 0, 1, and 2, **Figures 2A, B**). In contrast, significantly lower frequencies of CCCs/SARS-CoV-2 cross-reactive functional IFN-γ-producing CD8^+^ T cells were detected in the two groups of unvaccinated severely ill symptomatic COVID-19 patients (i.e., severity 3 and 4, mean SFCs < 50) and the unvaccinated COVID-19 patients with fatal outcomes (i.e., severity 5, mean SFCs < 25).

Out of the 27 CD8^+^ T-cell epitopes, there was a significant positive linear correlation between CD8^+^ T-cell responses specific to 22 epitopes and little to no severe COVID-19 disease (**Figures 2A, B**). For these 22 epitopes, the Pearson correlation coefficients (R) ranged from −0.8314 to −0.9541, and slopes (S) of the best-fitted lines comprised between −14.36 and −52.81. For the remaining five epitopes (ORF1ab_2210–2218_, ORF1ab_3013–3021_, ORF1ab_5470–5478_, S_691–699_, and S_976–984_), no significant linear correlation was observed. Nonetheless, among these five epitopes, the slope for ORF1ab_2210–2218_, ORF1ab_3013–3021_, and ORF1ab_5470–5478_ was comparatively less negative (S > −10) (**Figures 2A, C** and **Supplementary Figure 5**). Additionally, although we could not establish any significant linear correlation between S_691–699_ and S_976–984_ epitope-specific CD8^+^ T-cell responses and disease severity, more complex (non-linear) associations might exist. For example, the magnitude of the S_976–984_-specific IFN-γ-producing CD8^+^ T-cell response followed a clear downside trend, as the disease severity increased in severely ill symptomatic COVID-19 patients and patients with fatal outcomes (i.e., severity 3–5) (**Figures 2A, C** and **Supplementary Figure 5**).

Taken together, these results demonstrate that, like SARS-CoV-2-specific CD4^+^ T cells, an overall higher magnitude of CCCs/SARS-CoV-2 cross-reactive CD8^+^ T-cell responses were present in asymptomatic COVID-19 patients who never presented any COVID-19 symptoms, despite being infected. In contrast, a lower magnitude of CCCs/SARS-CoV-2 cross-reactive CD8^+^ T-cell responses was detected in severely ill COVID-19 patients and patients with fatal COVID-19 outcomes. These observations also highlight the importance of rapidly mounting strong CCCs/SARS-CoV-2 cross-reactive CD8^+^ T-cell responses, directed toward structural, non-structural, and accessory protein antigens, for protection against symptomatic and fatal Infections in unvaccinated COVID-19 patients.

### A broad lymphopenia, leukocytosis, and low frequencies of CD4^+^ and CD8^+^ T cells specific to highly conserved CCCs/SARS-CoV-2 cross-reactive epitopes are present in unvaccinated severely ill symptomatic COVID-19 patients

**Figure 3:**
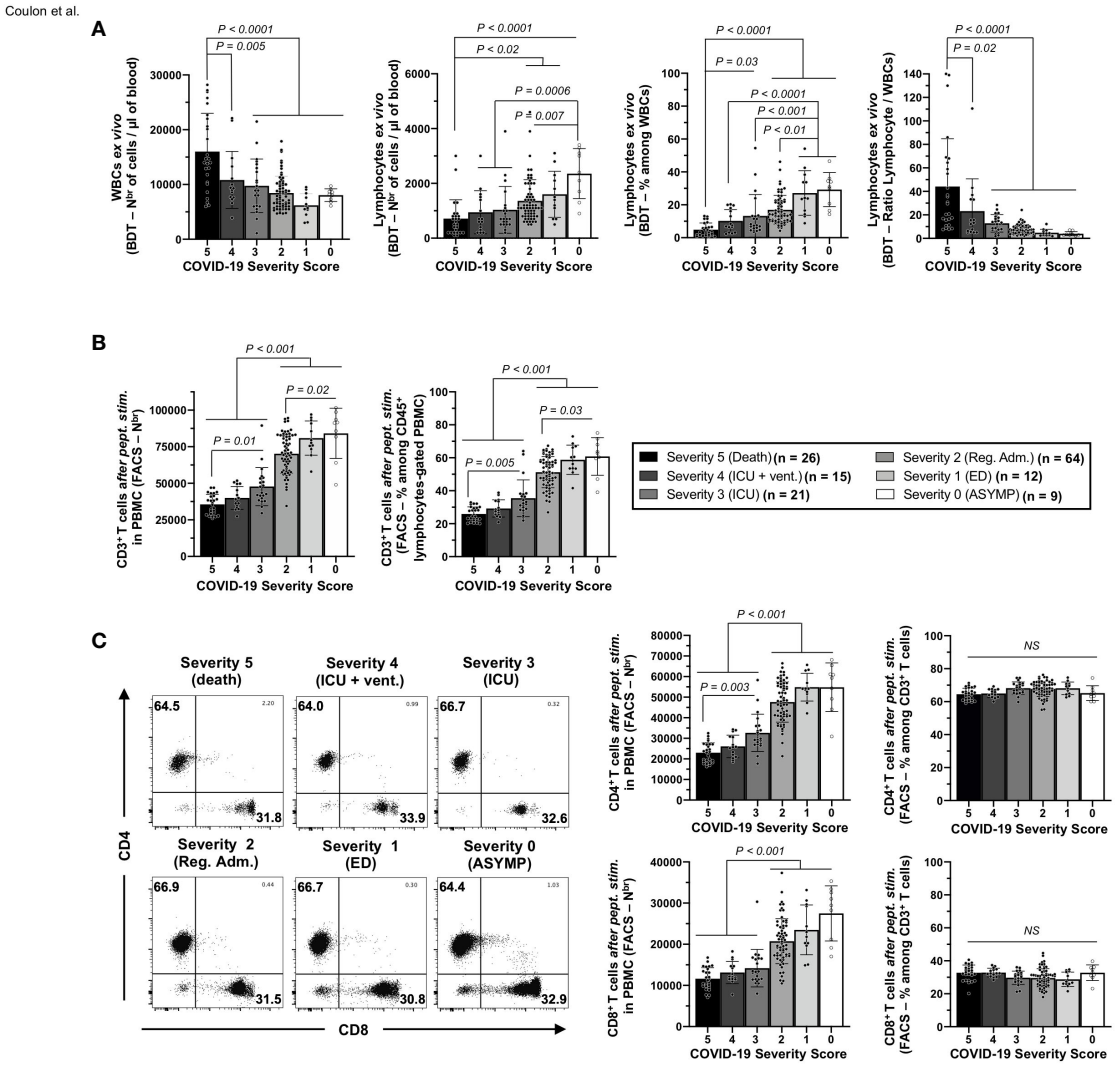
Frequencies of white blood cells, lymphocytes, and CD3^+^/CD4^+^/CD8^+^ T cells in the blood of unvaccinated COVID-19 patients with various degrees of disease severity. **(A)** numbers of white blood cells (WBCs) and total lymphocytes per µl of blood (left two panels) and percentages and ratios of total lymphocytes among WBCs (right two panels) measured *ex vivo* by blood differential test (BDT) in unvaccinated COVID-19 patients with various degrees of disease severity (*n* = 147). **(B)** Averages/means of numbers and frequencies of CD3^+^ T cells and **(C)** of total CD4^+^, and CD8^+^ T cells measured by flow cytometry from COVID-19 patients’ PBMCs with various severity scores after 72 h of stimulation with a pool of 16 CD4^+^ and 27 CD8^+^ CCCs/SARS-CoV-2 cross-reactive epitope peptides. The right panels show representative dot plots from patients with disease severity scores from 0 to 5. Data are expressed as the mean ±SD. Results are representative of two independent experiments and were considered statistically significant at *p* ≤ 0.05 (one-way ANOVA).

We next determined whether the low magnitudes of CCCs/SARS-CoV-2 cross-reactive CD4^+^ and CD8^+^ T-cell responses detected in unvaccinated severely ill and fatal COVID-19 patients was a result of an overall deficit in the frequencies of total CD4^+^ and CD8^+^ T cells. Using a blood differential test (BDT), we compared the absolute numbers of white blood cells (WBCs) and blood-derived lymphocytes, *ex vivo*, in the unvaccinated COVID-19 patients (**Figure 3A**).

A significant increase in the numbers of WBCs was detected in unvaccinated COVID-19 patients with fatal outcomes, (i.e., patients with severity 5, ∼1.5- to ∼2.6-fold) when compared with all the remaining five groups of unvaccinated COVID-19 patients (i.e., patients with severity 0, 1, 2, 3, and 4; *p* ≤ 0.02, **Figure 3A**—left panel). However, significantly lower absolute numbers of total lymphocytes were detected in the blood of unvaccinated COVID-19 patients with fatal outcomes (i.e., patients with severity 5) compared to unvaccinated COVID-19 patients with mild disease (i.e., patients with severity 1 and 2: ∼1.9- to ∼2.3-fold decrease—*p* < 0.02) or to asymptomatic patients with no disease (i.e., patients with severity 0: ∼3.3-fold decrease—*p* < 0.0001) (**Figure 3A**—second panel from left). As a result, the more severe the disease, the lower the percentage of blood-derived lymphocytes within WBCs (**Figure 3A**—third panel from left), and the lower the ratio of lymphocyte/WBCs (**Figure 3A**—fourth panel from left).

Overall, these results indicate that unvaccinated severely ill COVID-19 patients and unvaccinated COVID-19 patients with fatal outcomes not only had a general leukocytosis but also lymphopenia, which developed as early as 4.8 days after reporting their first symptoms or their first PCR-positive test.

Furthermore, we found a significant CD3^+^ T-cell lymphopenia positively associated with the onset of severe disease in unvaccinated COVID-19 patients (**Figure 3B**). On average, the two groups of unvaccinated severely ill COVID-19 patients and unvaccinated COVID-19 patients with fatal outcomes (i.e., patients with severity 3, 4, and 5) had a ∼1.9-fold decrease in absolute number of CD3^+^ T cells compared to three groups of unvaccinated asymptomatic COVID-19 patients with low to no severe disease (i.e., patients with severity 0, 1, and 2, **Figure 3B**, *p* < 0.001). Similarly, the numbers of total CD4^+^ and CD8^+^ T cells within CD3^+^-gated cells were reduced early in the two groups of unvaccinated severely ill COVID-19 patients and unvaccinated COVID-19 patients with fatal outcomes (i.e., patients with severity 3, 4, and 5) compared to the three groups of unvaccinated asymptomatic COVID-19 patients with low to no severe disease (**Figure 3C**—left column graph).

**Figure 4:**
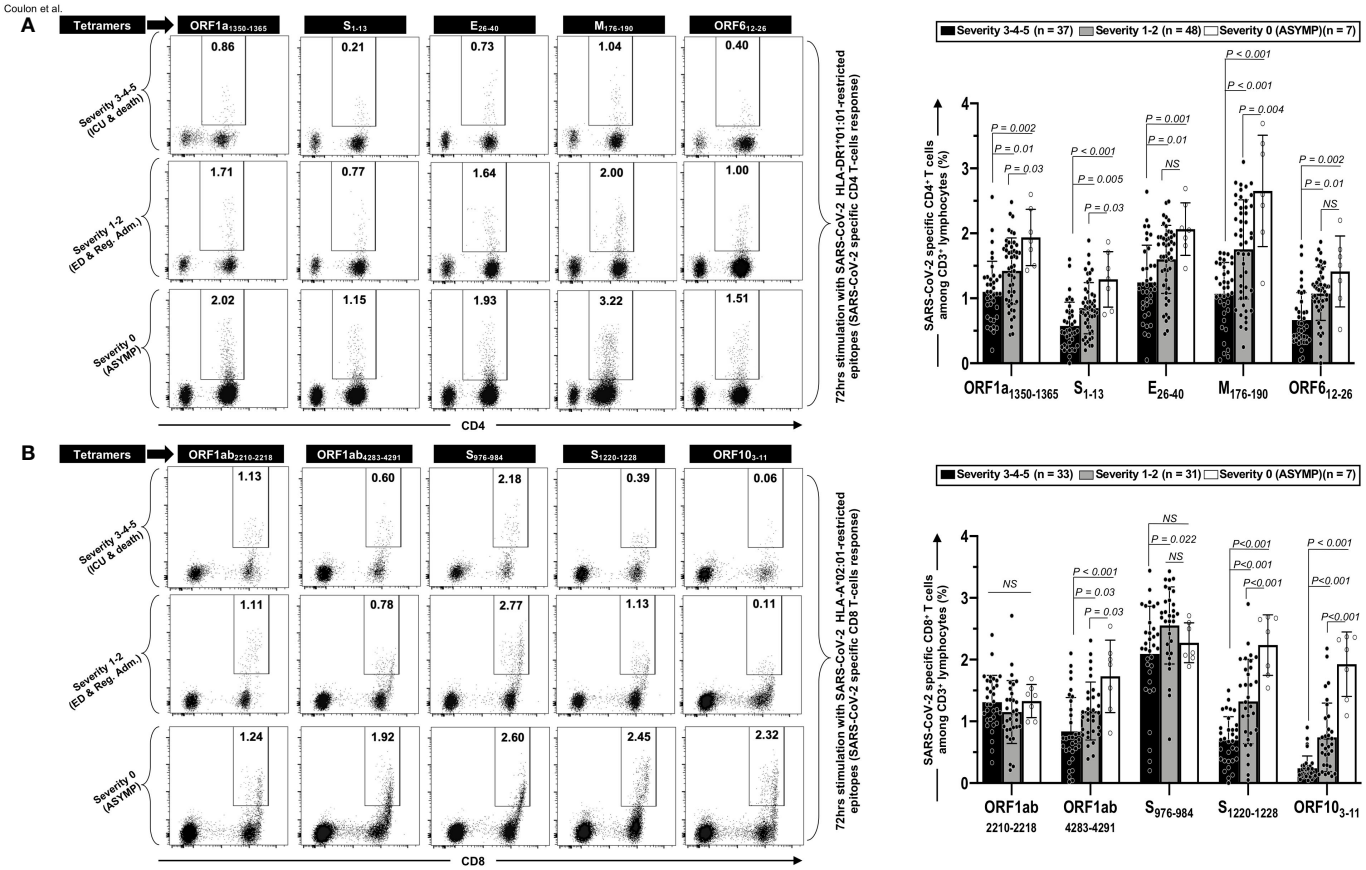
Frequencies of CCCs/SARS-CoV-2 cross-reactive CD4^+^ and CD8^+^ T cells in unvaccinated COVID-19 patients with various degrees of disease severity. PBMCs from HLA-DRB1*01:01-positive (*n* = 92) **(A)** or HLA-A*02:01-positive (*n* = 71) **(B)** unvaccinated COVID-19 patients with various degrees of disease severity were isolated and stimulated for 72 h with 10 μg/ml of indicated CCCs/SARS-CoV-2 cross-reactive CD4^+^ and CD8^+^ epitope peptides. The induced CD4^+^ and CD8^+^ T cells were then stained and analyzed by flow cytometry. The indicated epitope peptides were chosen among the CCCs/SARS-CoV-2 cross-reactive 16 CD4^+^ and 27 CD8^+^ epitope peptides based on tetramer availability. Panel **(A)** shows representative dot plots (left panels) and average frequencies of CCCs/SARS-CoV-2 cross-reactive CD4^+^ T cells (right panel) detected in three representatives COVID-19 patients, each falling into one of three groups of disease category: the unvaccinated asymptomatic COVID-19 patients (ASYMP, severity score 0), unvaccinated COVID-19 patients who developed mild to moderate disease (severity scores 1 and 2), and unvaccinated severely ill COVID-19 patients and unvaccinated patients with fatal COVID-19 outcomes (severity scores 3–5). Panel **(B)** shows representative dot plots (left panels) and average frequencies of CCCs/SARS-CoV-2 cross-reactive CD8^+^ T cells (right panel) detected in three representatives of COVID-19 patients and in panel **(A)**. Data are expressed as the mean ± SD. Results are representative of two independent experiments and were considered statistically significant at *p* ≤ 0.05 (one-way ANOVA).

Finally, we determined the frequencies of SARS-CoV-2-specific CD4^+^ and CD8^+^ T cells following a 72-h *in vitro* stimulation with individual CD4^+^ and CD8^+^ T epitope peptides (as illustrated in **Supplementary Figure 2**). We used tetramers specific to five highly conserved CCCs/SARS-CoV-2 cross-reactive DRB1*01:01-restricted CD4^+^ T-cell epitopes ORF1a_1350–1365_, S_1–13_, E_26–40_, M_176–190_, and ORF6_12–26_ (**Figure 4A**) and five highly conserved CCCs/SARS-CoV-2 cross-reactive HLA-A*02:01-restricted CD8^+^ T-cell epitopes Orf1ab_2210–2218_, Orf1ab_4283–4291_, S_976–984_, S_1220–1228_, and ORF10_3–11_ (**Figure 4B**).

We found a significant decrease in the frequencies of CD4^+^ T cells specific to all the five highly conserved CCCs/SARS-CoV-2 cross-reactive DRB1*01:01-restricted epitopes in the three groups of unvaccinated severely ill COVID-19 and unvaccinated COVID-19 patients with fatal outcomes (i.e., patients with severity 3, 4, and 5) compared to the remaining three groups of unvaccinated COVID-19 patients with low to no severe disease (i.e., patients with severity 1, 2—*p* ≤ 0.01) and to unvaccinated asymptomatic COVID-19 patients (severity 0—*p* ≤ 0.002) (**Figure 4A**). Similarly, we found a significant decrease in the frequencies of CD8^+^ T cells specific to three out the five highly conserved CCCs/SARS-CoV-2 cross-reactive HLA-A*02:01-restricted CD8^+^ T-cell epitopes (Orf1ab_4283–4291_, S_1220–1228_, and ORF10_3–11_) in the three groups of unvaccinated severely ill COVID-19 and unvaccinated COVID-19 patients with fatal outcomes (i.e., patients with severity 3, 4, and 5) compared to unvaccinated COVID-19 patients with low to no severe disease (i.e., patients with severity 1 and 2—*p* ≤ 0.03) and to unvaccinated asymptomatic COVID-19 patients (severity 0—*p* < 0.001) (**Figure 4B**). In contrast, similar frequencies of EBV BMLF-1_280–288_-specific CD8^+^ T cells were detected across the six groups of unvaccinated COVID-19 patients, regardless of disease severity, indicating that the decrease in the frequencies of T cells in severely ill COVID-19 patients specifically affected highly conserved and CCCs/SARS-CoV-2 cross-reactive T cells (**Supplementary Figure 3A**).

Taken together, our findings demonstrate that, compared to asymptomatic COVID-19 patients who presented with little to no disease, the severely ill patients and patients with fatal COVID-19 outcomes showed the following: (i) a broad and early lymphopenia (and leukocytosis), (ii) a decrease of bulk CD3^+^ T-cell lymphocytes number (equally affecting CD4^+^ and CD8^+^ T cells), and (iii) a reduction in CD4^+^ and CD8^+^ T cells specific to highly conserved CCCs/SARS-CoV-2 cross-reactive epitopes from structural, non-structural, and accessory protein antigens.

### Unvaccinated severely ill COVID-19 patients present high frequencies of phenotypically and functionally exhausted CCCs/SARS-CoV-2 cross-reactive CD4^+^ and CD8^+^ T cells, detected both *ex vivo* and *in vitro*

We next compared the phenotype and function of CD4^+^ and CD8^+^ T cells specific to CCCs/SARS-CoV-2 cross-reactive epitopes in unvaccinated asymptomatic COVID-19 patients, with little to no disease, versus the unvaccinated severely ill COVID-19 patients and the unvaccinated COVID-19 patients with fatal outcomes.

**Figure 5:**
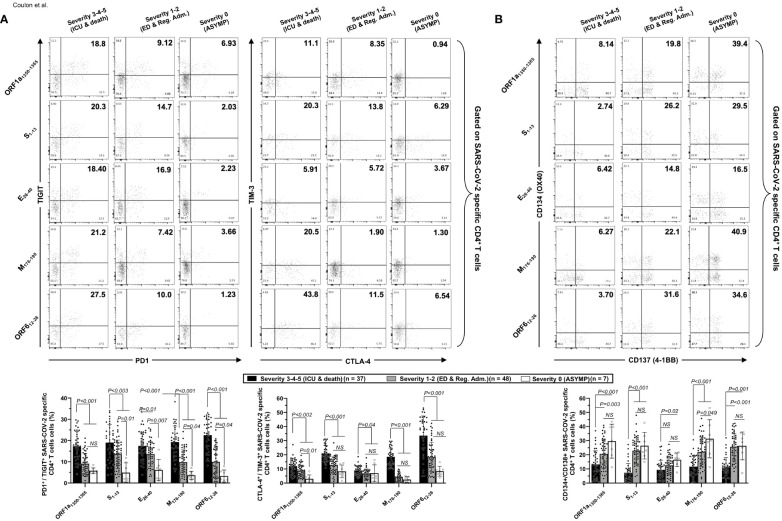
Co-expression of exhaustion and activation markers on CCCs/SARS-CoV-2 cross-reactive CD4^+^ T cells from unvaccinated COVID-19 patients with various degrees of disease severity. PBMCs from HLA-DRB1*01:01-positive unvaccinated COVID-19 patients with various degrees of disease severity were isolated and stimulated for 72 h with 10 μg/ml of five CCCs/SARS-CoV-2 cross-reactive CD4^+^ T-cell epitope peptides. The induced CD4^+^ T cells were then stained and analyzed by flow cytometry for the frequency of tetramer-specific CD4^+^ cells co-expressing exhaustion and activation markers. Panel **(A)** shows representative dot plots (upper panels) and average (lower panels) frequencies of CCCs/SARS-CoV-2 cross-reactive CD4^+^ T cells expressing exhaustion markers PD1/TIGIT and TIM-3/CTLA-4 detected in three representative groups of unvaccinated COVID-19 patients with various degrees of disease severity. Panel **(B)** shows representative dot plots (upper panels) and average (lower panels) frequencies of CCCs/SARS-CoV-2 cross-reactive CD4^+^ T cells expressing activation markers (AIMs) CD134/CD137 detected in three representative groups of unvaccinated COVID-19 patients with various degrees of disease severity. Results are representative of two independent experiments, and data are expressed as the mean ± SD and were considered statistically significant at *p* ≤ 0.05 calculated using one-way ANOVA.

**Figure 6:**
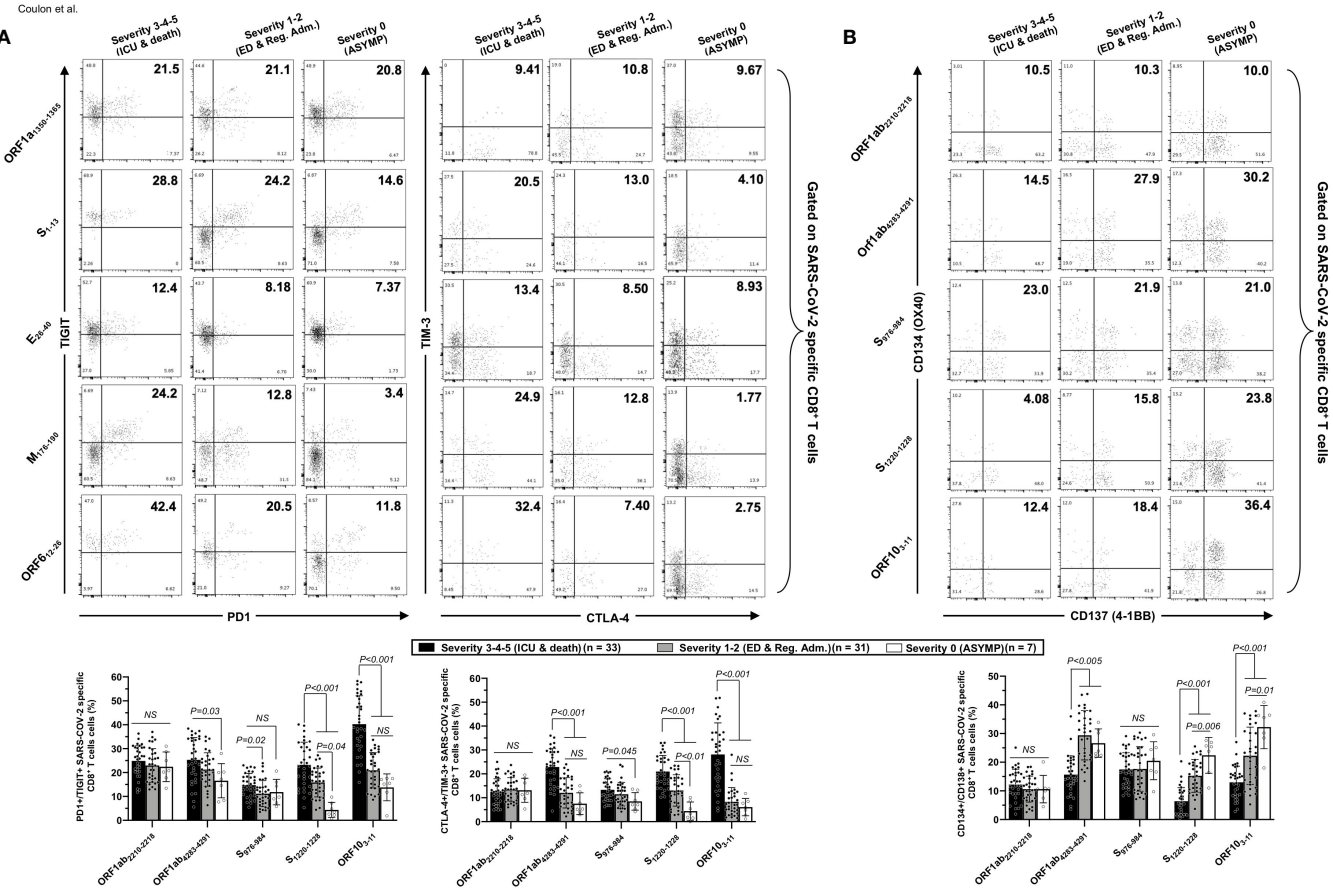
Co-expression of exhaustion and activation markers on CCCs/SARS-CoV-2 cross-reactive CD8^+^ T cells from unvaccinated COVID-19 patients with various degrees of disease severity. PBMCs from HLA-A*02:01-positive unvaccinated COVID-19 patients with various degrees of disease severity were isolated and stimulated for 72 h with 10 μg/ml of five CCCs/SARS-CoV-2 cross-reactive CD8^+^ T-cell epitope peptides. The induced CD8^+^ T cells were then stained and analyzed by flow cytometry for the frequency of tetramer-specific CD8^+^ cells co-expressing exhaustion and activation markers. Panel **(A)** shows representative dot plots (upper panels) and average frequencies of CCCs/SARS-CoV-2 cross-reactive CD8^+^ T cells (lower panel) expressing exhaustion markers PD1/TIGIT and TIM-3/CTLA-4 detected in three representative groups of unvaccinated COVID-19 patients with various degrees of disease severity. Panel **(B)** shows representative dot plots (upper panels) and average frequencies of CCCs/SARS-CoV-2 cross-reactive CD8^+^ T cells (lower panel) expressing activation markers (AIMs) CD134/CD137 detected in three representative groups of unvaccinated COVID-19 patients with various degrees of disease severity. Results are representative of two independent experiments, and data are expressed as the mean ± SD and were considered statistically significant at *p* ≤ 0.05 (one-way ANOVA).

Co-expression of four main exhaustion markers (PD-1, TIM3, TIGIT, and CTLA4) and two activation markers (AIMs) CD137 (4-1BB) and CD134 (OX40) were compared using FACS and tetramers specific to five highly conserved CCCs/SARS-CoV-2 cross-reactive DRB1*01:01-restricted CD4^+^ T-cell epitopes, ORF1a_1350–1365_, S_1–13_, E_26–40_, M_176–190_, and ORF6_12–26_ both *in vivo* (**Figure 5**) and *ex vitro* (**Supplementary Figure 6**) and five highly conserved CCCs/SARS-CoV-2 cross-reactive HLA-A*02:01-restricted CD8^+^ T-cell epitopes, Orf1ab_2210–2218_, Orf1ab_4283–4291_, S_976–984_, S_1220–1228_, and ORF10_3–11_ both *in vitro* (**Figure 6**) and *ex vivo* (**Supplementary Figure 6**).

We detected significantly higher frequencies of phenotypically exhausted SARS-CoV-2-specific CD4^+^ T cells in unvaccinated symptomatic COVID-19 patients with high severity scores (i.e., patients with severity 3, 4, and 5) compared to unvaccinated asymptomatic COVID-19 patients (i.e., patients with severity 0) (**Figure 5A**—up to ∼6.9-fold increase for ORF6_12–26_-specific PD-1^+^TIGIT^+^CD4^+^ T cells and up to ∼7.8-fold increase for M_176–190_-specific TIM-3^+^CTLA-4^+^CD4^+^ T cells). Similarly, there were significantly higher frequencies of phenotypically exhausted CD8^+^ T cells in unvaccinated severely ill COVID-19 and patients with fatal outcomes compared to unvaccinated asymptomatic COVID-19 patients (**Figure 6A**—up to ∼3.6-fold increase for S_1220–1228_-specific PD-1^+^TIGIT^+^CD8^+^ T cells and up to ∼4.6-fold increase for S_1220–1228_- and ORF10_3-11_-specific TIM-3^+^CTLA-4^+^CD8^+^ T cells). Overall, except for Orf1ab_2210–2218_- and S_976–984_-specific-CD8^+^ T cells, the unvaccinated severely ill and fatal patients (i.e., patients with severity 3, 4, and 5) had significantly higher frequencies of exhausted CD8^+^ T cells co-expressing PD-1^+^TIGIT^+^ or TIM-3^+^CTLA-4 compared to unvaccinated asymptomatic COVID-19 patients with little to no disease (i.e., patients with severity 0, 1, and 2). The Orf1ab_2210–2218_- and S_976–984_-specific-CD8^+^ T cells did not demonstrate any significantly higher phenotypic exhaustion in unvaccinated severely ill COVID-19 patients. We confirmed *ex vivo* that the unvaccinated severely ill and fatal patients (i.e., patients with severity 3, 4, and 5) had significantly higher frequencies of exhausted CD8^+^ T cells co-expressing PD-1^+^TIGIT^+^ or TIM-3^+^CTLA-4 compared to unvaccinated asymptomatic COVID-19 patients with little to no disease (i.e., patients with severity 0, 1, and 2, *p* < 0.05, **Supplementary Figure 6**).

Accordingly, we also detected low frequencies of functional CD134^+^CD137^+^CD4^+^ T cells (**Figure 5B**) and low frequencies of functional CD134^+^CD137^+^CD8^+^ T cells (**Figure 6B**) in unvaccinated severely ill COVID-19 patients and unvaccinated patients with fatal COVID-19 outcomes. This applied to CD134^+^CD137^+^CD4^+^ T cells specific to CCCs/SARS-CoV-2 cross-reactive epitopes from all five structural and non-structural proteins and to CD134^+^CD137^+^CD8^+^ T cells specific to three out of five CCCs/SARS-CoV-2 cross-reactive epitopes from structural and non-structural proteins.

As expected, no differences were observed in phenotypic and functional exhaustion of EBV BMLF-1_280–288_-specific CD8^+^ T cells across the six groups of COVID-19 patients with various disease severities (**Supplementary Figure 3B**), suggesting that the exhaustion of CD4^+^ and CD8^+^ T cells in severely ill COVID-19 patients and to patients with fatal COVID-19 outcomes was specific to CCCs/SARS-CoV-2 cross-reactive epitopes.

Altogether, these results (i) indicate that phenotypic and functional exhaustion of CD4^+^ and CD8^+^ T cells, detected both *ex vivo* and *in vitro*, specific to highly conserved and CCCs/SARS-CoV-2 cross-reactive epitopes from both structural and non-structural antigens was associated with symptomatic and fatal infections in unvaccinated COVID-19 patients and (ii) suggest the importance of functional CCCs/SARS-CoV-2 cross-reactive CD4^+^ and CD8^+^ T cells, directed toward structural, non-structural, and accessory protein antigens, for protection against symptomatic and fatal infections in unvaccinated COVID-19 patients.

### Higher rates of co-infection with alpha common cold coronavirus 229E present unvaccinated asymptomatic COVID-19 patients

**Figure 7:**
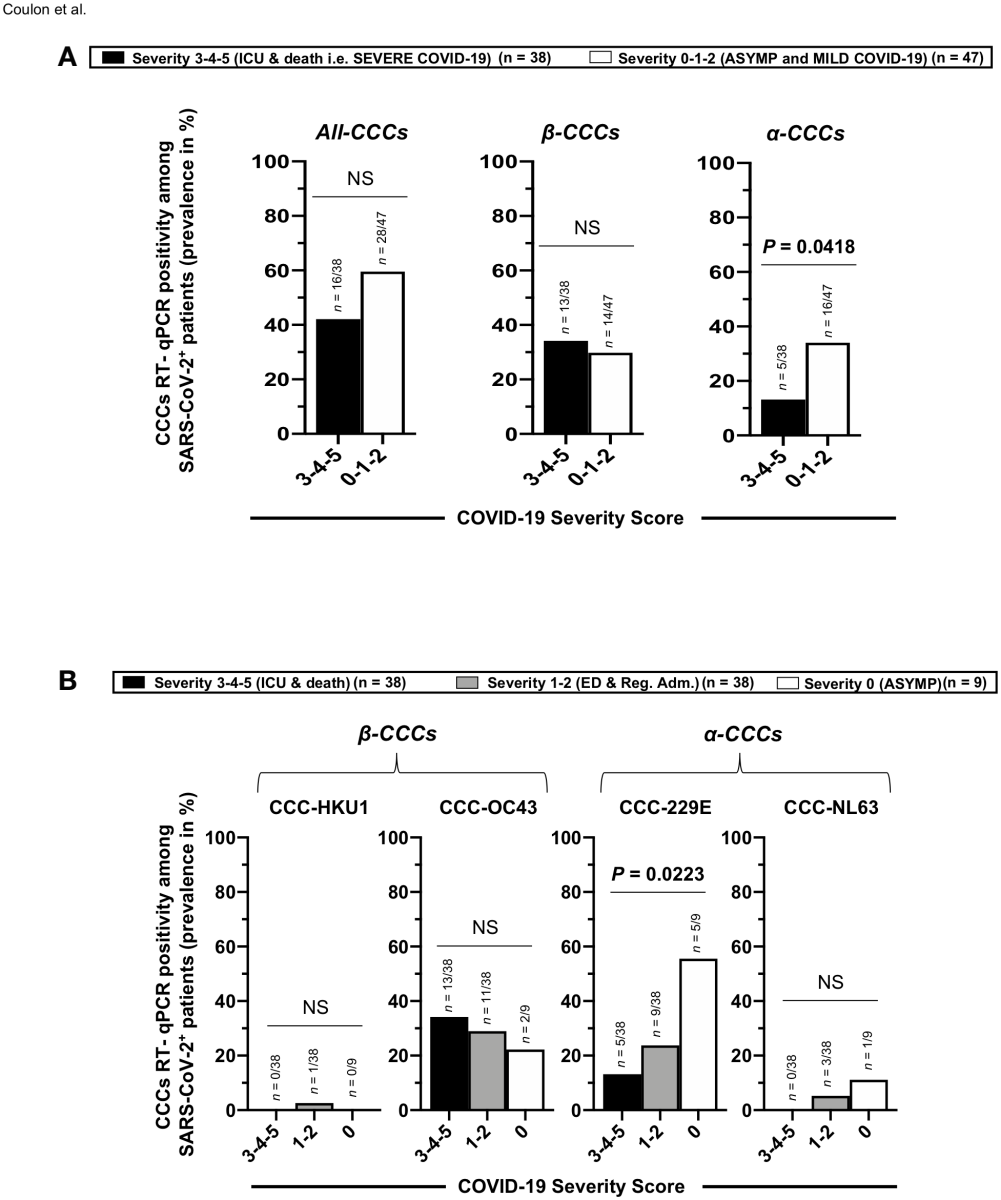
Rates (frequency) of co-infection with seasonal common cold coronavirus species α-CCC-NL63, α-CCC-229E, β-CCC HKU1, and β-CCC-OC43 in unvaccinated COVID-19 patients with various degrees of disease severity. Four major human common cold coronaviruses species, CCC-HKU1, CCC-OC43, CCC-229E, and CCC-NL63, were detected using RT-PCR in the nasopharyngeal swabs of COVID-19 patients (*n* = 85, first column) who developed various disease severity. Panel **(A)** shows all four α-CCCs and β-CCCs species (left panel), β-CCC species alone (middle panel), and α-CCC species alone (right panel), detected in unvaccinated severely ill COVID-19 patients and unvaccinated patients with fatal COVID-19 outcomes (severity scores 3–4–5) vs. unvaccinated COVID-19 patients who developed no, mild, and moderate disease (severity score 1–2–3). **(B)** The rate (%) of co-infection with each one of the four major species, CCC-HKU1, CCC-OC43, CCC-229E, and CCC-NL63, detected in unvaccinated severely ill COVID-19 patients and unvaccinated patients with fatal COVID-19 outcomes (severity scores 3–4–5), in unvaccinated COVID-19 patients who developed mild to moderate disease (severity score 1–2), and in unvaccinated asymptomatic COVID-19 patients (severity score 0). The *p*-values calculated using the Chi-squared test compare the rate (%) of co-infection with each CCC species between unvaccinated COVID-19 patients with various degrees of disease severity. Results are representative of two independent experiments, and data are expressed as the mean ± SD and were considered statistically significant at *p* ≤ 0.05 calculated using Fisher’s exact test.

We next compared the co-infection with each of the four main and seasonal α and β CCCs (i.e., α-CCC-NL63, α-CCC-229E, β-CCC-HKU1, and β-CCC-OC43) in a cohort of 85 unvaccinated COVID-19 patients divided into six groups based on the severity of COVID-19 symptoms, as above (i.e., patients with severity 5 to severity 0, **Figures 7A, B**). Using RT-PCR performed on nasopharyngeal swab samples, we found co-infection with the α-CCC species to be more common with significantly higher rates in the asymptomatic COVID-19 patients (i.e. unvaccinated naturally protected from severe symptoms) compared to severely ill COVID-19 patients and to unvaccinated patients with fatal outcomes (i.e., unvaccinated that were not naturally protected from severe symptoms) (**Figure 7A**—right panel; ∼2.6-fold increase in groups 1–2–3 versus groups 4–5–6 of disease severity; *p* = 0.0418 calculated with Fisher’s exact test). Co-infection with the CoV-229E α-CCC species was more common with significantly higher rates in the unvaccinated asymptomatic COVID-19 patients compared to unvaccinated severely ill COVID-19 patients and unvaccinated patients with fatal outcomes (**Figure 7B**, right panels: ∼4.2-fold increase between unvaccinated asymptomatic COVID-19 patients and unvaccinated severely ill COVID-19 patients (i.e., patients with severity of 4–5–6; *p* = 0.0223). However, there was no significant difference in the rates of co-infection with β-CCC species (nor with any of the four CCC species) across all six groups of COVID-19 patients with various severity symptoms (**Figure 7A**, central and left panels, and **Figure 7B**, left two panels).

**Figure 8:**
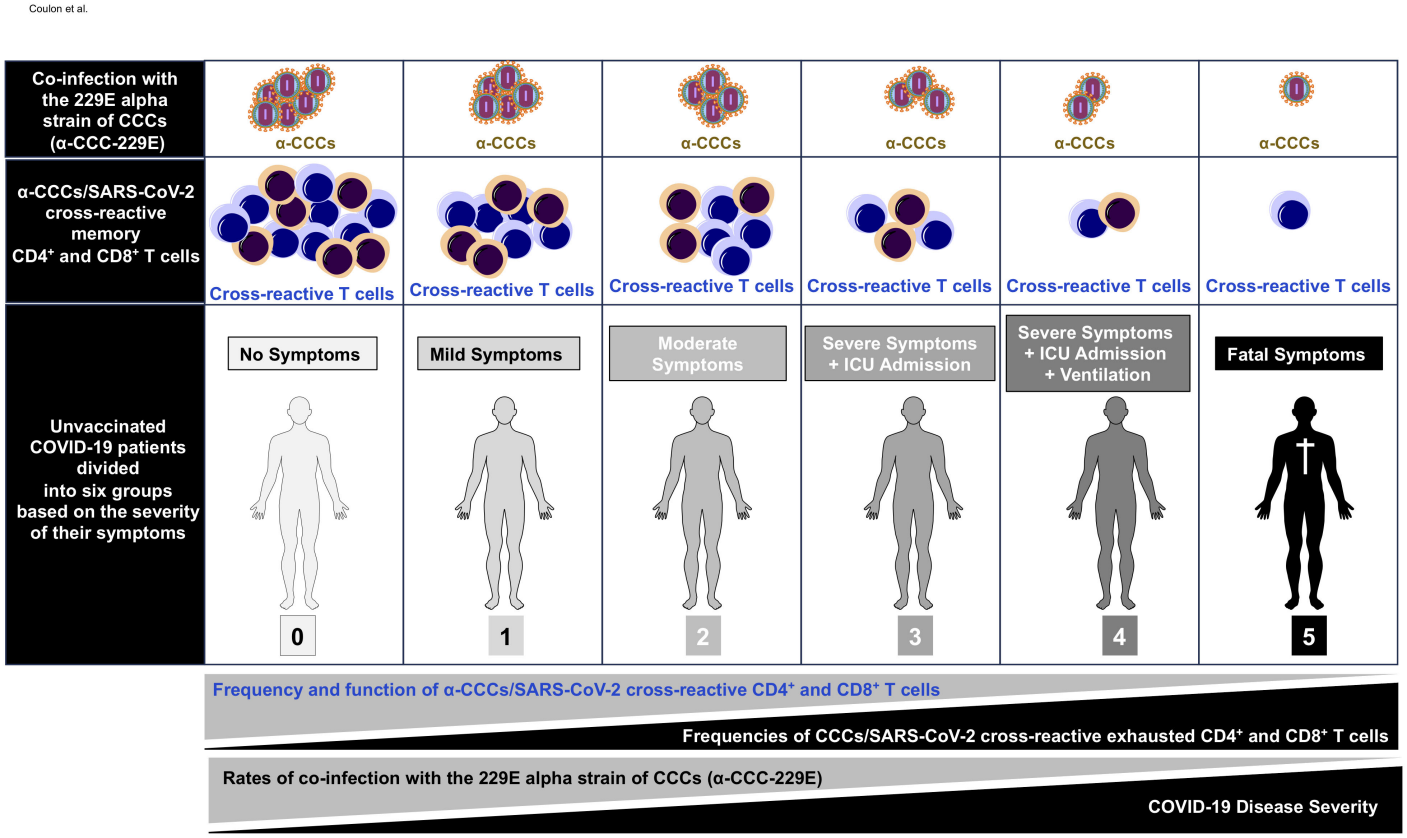
Illustration showing higher frequencies of common cold coronavirus/SARS-CoV-2 cross-reactive CD4^+^ and CD8^+^ T cells detected in unvaccinated asymptomatic COVID-19 patients is associated with higher rates of co-infection with alpha common cold coronavirus strain 229E (α-CCC-229E). The first row shows increasing copies of α-CCC-229E detected in unvaccinated asymptomatic COVID-19 patients compared to unvaccinated symptomatic COVID-19 patients. The middle row shows increasing numbers of common cold coronavirus/SARS-CoV-2 cross-reactive CD4^+^ and CD8^+^ memory T cells detected in unvaccinated asymptomatic COVID-19 patients compared to unvaccinated symptomatic COVID-19 patients. The bottom row shows symptoms detected in unvaccinated COVID-19 patients with symptoms increasing from severity 0 in asymptomatic COVID-19 patients (left) to severity 5 in COVID-19 patients with fatal COVID-19 outcomes (right) as detailed in *Materials and methods*.”.

As illustrated in **Figure 8**, these results indicate that (i) compared to severely ill COVID-19 patients and patients with fatal COVID-19 outcomes, the asymptomatic COVID-19 patients presented significantly higher rates of co-infection with the α-CCC species, and with the 229E of α-CCCs, in particular and (*ii*) suggest that co-infection with the α species of CCCs (particularly the 229E species of α-CCCs, but not the β species) was associated with the natural protection from symptomatic and fatal infections in unvaccinated COVID-19 patients with yet-to-be-determined mechanisms(s).

### High frequencies of α-CCCs/SARS-CoV-2 cross-reactive memory CD4^+^ and CD8^+^ T cells are associated with natural protection from symptomatic and fatal infections in unvaccinated COVID-19 patients

Next, we determined whether (i) the higher rates of co-infection with α-CCC species observed in the unvaccinated asymptomatic COVID-19 patients were associated with high frequencies of CCCs/SARS-CoV-2 cross-reactive CD4^+^ and CD8^+^ T cells detected in these asymptomatic COVID-19 groups and (ii) the high frequencies of α-CCCs/SARS-CoV-2 cross-reactive epitope-specific CD4^+^ and CD8^+^ T cells were associated with fewer symptoms observed in unvaccinated COVID-19 patients. To this end, we determined the percentage of unvaccinated asymptomatic COVID-19 patients, unvaccinated severely ill COVID-19, and unvaccinated patients with fatal outcomes who presented significant IFN-γ^+^CD4^+^ and IFN-γ^+^CD8^+^ T-cell responses (i.e., IFN-γ^-^ELISpot SFCs > 50) specific to α-CCCs/SARS-CoV-2 cross-reactive epitopes.

Significantly higher percentages of unvaccinated asymptomatic COVID-19 patients with significant IFN-γ^+^CD4^+^ and IFN-γ^+^CD8^+^ T-cell responses specific to α-CCCs/SARS-CoV-2 cross-reactive epitopes were observed (*p* < 0.001). Similarly, the α-CCCs/SARS-CoV-2 cross-reactive epitopes were strongly cross-recognized by IFN-γ^+^CD4^+^ T cells (SFCs>50) and CD8^+^ T cells from both unexposed pre-pandemic healthy individuals (UPPHI) and unvaccinated asymptomatic COVID-19 patients. In contrast, low frequencies of unvaccinated severely ill COVID-19 patients and unvaccinated patients with fatal outcomes significant IFN-γ^+^CD4^+,^ and IFN-γ^+^CD8^+^ T-cell responses specific to α-CCCs/SARS-CoV-2 cross-reactive epitopes (*p* < 0.001) (**Supplementary Table 4**). We also found that unexposed pre-pandemic healthy individuals (UPPHI) who were never exposed to SARS-CoV-2 presented α-CCCs/SARS-CoV-2 cross-reactive IFN-γ^+^CD4^+^ and IFN-γ^+^CD8^+^ T cells specific to highly conserved SARS-CoV-2 epitopes (**Supplementary Figure 7**), confirming our report and others’ previous reports (1, 16, 21, 41, 43, 44).

Taken together, these results demonstrate that, compared to low proportions of severely ill COVID-19 patients and patients with fatal outcomes, significant proportions of both unvaccinated asymptomatic COVID-19 patients and unexposed pre-pandemic healthy individuals (UPPHI) presented significant α-CCCs/SARS-CoV-2 strong cross-reactive CD4^+^ and CD8^+^ T-cell responses. These findings suggest a crucial role of functional α-CCCs/SARS-CoV-2 cross-reactive memory CD4^+^ and CD8^+^ T cells, induced following previous α-CCC seasonal exposures, in protection against subsequent severe symptomatic SARS-CoV-2 infection, as illustrated in **Figure 8**.

### Cross-reactive CD4^+^ and CD8^+^ T-cell epitopes from α-CCCs and SARS-CoV-2 that present high similarity and identity are associated with natural protection from symptomatic and fatal infections in unvaccinated COVID-19 patients

Using both the Multiple Sequences Alignments (MSA) and the Epitope Conservancy Tool (ECT) algorithms and software, we determined the identity (%id) and the similarity scores (S^s^) of cross-reactive CD4^+^ and CD8^+^ T-cell epitopes, between the four major CCC species (α-hCCC-NL63, α-hCCC-229E, and β-hCCC-HKU1, β-hCCC-OC43), on the one hand, and SARS-CoV-2, on the other hand, as described in *Materials and methods* (58), (**Table 2** and **Supplementary Tables 1**-**3**).

Of the 16 highly conserved CD4^+^ T-cell epitopes (**Supplementary Figure 8**), the ORF1ab_5019–5033_ epitope was highly conserved (%id ≥ 67%) and highly similar (S^S^ ≥ 0.8) between SARS-CoV-2 and the two β-CCC species (β-CCC-HKU1 and β-CCC-OC43), while the ORF1ab_6088–6102_ epitope was highly conserved between SARS-CoV-2 and both β-CCC-HKU1 and α-CCC-NL63 species (**Table 2** and **Supplementary Tables 1**-**3**). Five out of the 27 CD8^+^ T-cell epitopes (ORF1ab_3013–3021_, ORF1ab_6749–6757_, S_958–966_, E_20–28_, and M_52–60_) were highly conserved (% id ≥67%) and highly similar (S^S^ ≥ 0.8) between SARS-CoV-2 and the α-CCCs and/or β-CCC species. Specifically, the ORF1ab_3013–3021_ CD8^+^ T-cell epitope was highly conserved between SARS-CoV-2 and the two β-CCC species (β-CCC-HKU1 and β-CCC-OC43); the ORF1ab_6749–6757_ epitope was highly conserved between SARS-CoV-2 and all the four CCC species; the S_958–966_ epitope was highly conserved between SARS-CoV-2, the two β-CCC species, and the α-CCC-NL63 species; the E_20–28_ epitope was highly conserved between SARS-CoV-2 and the β-CCC-HKU1 species; and the M_52-60_ epitope was highly conserved between SARS-CoV-2, the two β-CCC species (β-CCC-HKU1 and β-CCC-OC43) and the α-CCC-229E species (**Supplementary Figure 9**, **Table 2**, and **Supplementary Tables 1**-**3**). While the E_20–28_ epitope was conserved (%id = 67%) between SARS-CoV-2 and α-CCC-NL63 species, it did not present significant similarity (**Table 2** and **Supplementary Tables 1**-**3**).

Next, we determined the corresponding NL63 peptide (S^S^ = 0.76). While the S_976–984_ epitope was conserved between SARS-CoV-2 and three CCC species (%id = 67%), it did not present significant similarity with the corresponding CCC peptides [β-CCC-HKU1 (S^S^=0.78), β-CCC-OC43 (S^S^=0.78) and α-CCC-NL63 (S^S^ = 0.73)]. Finally, while the S_2–10_ epitope was highly similar between SARS-CoV-2 and α-CCC-NL63 (S^S^ = 0.82), it was not significantly identical (id% = 56%) (**Table 2** and **Supplementary Tables 1**-**3**).

Next, we determined whether the CCCs/SARS-CoV-2-cross-reactive epitopes were cross-recognized preferentially by the CD4^+^ and CD8^+^ T cells from either unvaccinated asymptomatic COVID-19 patients, or unvaccinated severely ill COVID-19 patients and unvaccinated patients with fatal outcomes (**Supplementary Table 4**). No significant differences were detected when the slopes S of the SARS-CoV-2-specific CD4^+^ and CD8^+^ T-cell responses were applied towards epitopes that have no significant identity nor similarity to epitopes from the four CCCs. Significant differences were detected when the slopes S of the SARS-CoV-2-specific CD4^+^ and CD8^+^ T-cell responses were applied to epitopes that have significant identity and/or similarity to epitopes from at least one of the four CCCs (**Supplementary Table 4**). In contrast, SARS-CoV-2 CD4^+^ or CD8^+^ T cells cross-recognizing epitopes that are highly identical and similar exclusively in β-CCC species, but not in α-CCC species (i.e., epitopes ORF1ab_5019-5033_ and ORF1ab_3013-3021_), presented a significantly lower slope S (*p* = 0.04) (**Supplementary Table 4**). The ORF1ab_5019–5033_ and ORF1ab_3013–3021_ epitopes have slopes S close to 0 among all epitopes (**Supplementary Table 4**).

These data indicated that (i) CCCs/SARS-CoV-2-cross-reactive CD4^+^ or CD8^+^ T-cell epitopes that share high identity and similarity exclusively with the α-CCC species were cross-recognized mainly by CD4^+^ or CD8^+^ T cells from asymptomatic COVID -19 patients; (ii) in contrast, the CCCs/SARS-CoV-2-cross-reactive CD4^+^ or CD8^+^ T cell epitopes that share high identity and similarity exclusively with the β-CCC species were cross-recognized mainly by CD4^+^ or CD8^+^ T cells from severely ill symptomatic patients; and (iii) compared to severely ill COVID-19 patients and patients with fatal outcomes, the asymptomatic COVID-19 patients presented significantly higher frequencies of α-CCCs/SARS-CoV-2 cross-reactive CD4^+^ and CD8^+^ T cells. The findings suggest a crucial role of functional, poly-antigenic α-CCCs/SARS-CoV-2 cross-reactive memory CD4^+^ and CD8^+^ T cells, induced following previous α-CCC seasonal exposures, in protection against subsequent severe symptomatic SARS-CoV-2 infection.

## Discussion

Characterizing the underlying T-cell mechanisms associated with protection against COVID-19 severity in unvaccinated asymptomatic patients is a challenging task today, since most individuals have received at least one dose of COVID-19 vaccine (39). Only 15.2% of adults in the United States are unvaccinated (37, 38). This study is one of the few to comprehensively characterize the cross-reactive memory CD4^+^ and CD8^+^ T cells in unvaccinated symptomatic and asymptomatic COVID-19 patients. We compared the antigen specificity, frequency, phenotype, and function of CCCs/SARS-CoV-2 cross-reactive memory CD4^+^ and CD8^+^ T cells, cross-recognizing genome-wide conserved epitopes in a cohort of 147 unvaccinated COVID-19 patients, divided into six groups based on the severity of their symptoms. The findings demonstrate several relationships between antigen-specific T-cell responses and disease outcome. Specifically, severely ill symptomatic COVID-19 patients who required admission to intensive care units (ICUs) and patients with fatal COVID-19 outcomes, versus unvaccinated asymptomatic COVID-19 patients, displayed significantly (i) higher rates of co-infection with the 229E alpha species of CCCs (α-CCC-229E); (ii) higher frequencies of α-CCCs/SARS-CoV-2 cross-reactive functional memory CD134^+^CD137^+^CD4^+^ and CD134^+^CD137^+^CD8^+^ T cells, directed toward conserved epitopes from structural, non-structural, and accessory SARS-CoV-2 proteins; and (iii) lower frequencies of CCCs/SARS-CoV-2 cross-reactive and exhausted PD-1^+^TIM3^+^TIGIT^+^CTLA4^+^CD4^+^ and PD-1^+^TIM3^+^TIGIT^+^CTLA4^+^CD8^+^ T cells. These observations (i) support a crucial role for functional, poly-antigenic α-CCCs/SARS-CoV-2 cross-reactive memory CD4^+^ and CD8^+^ T cells, induced following previous α-CCC seasonal exposures, in protection against subsequent severe symptomatic SARS-CoV-2 infection and (ii) provide critical insights into developing broadly protective, multi-antigen, CD4^+^ and CD8^+^ T-cell-based, universal pan-Coronavirus vaccines capable of conferring cross-species protection.

The present comprehensive study of cross-reactive SARS-CoV-2 epitope-specific CD4^+^ and CD8^+^ T cells suggests that pre-pandemic exposure to seasonal α-CCC species, but not to β-CCC species, may have conferred protection from symptomatic COVID-19 infections by an as-yet-to-be-determined mechanism(s). It is likely that pre-existing CCCs/SARS-CoV-2 cross-reactive memory CD4^+^ and CD8^+^ T cells, induced in UPPHI by seasonal α-CCC species, cross-recognized protective SARS-CoV-2 epitopes. These data are consistent with previous studies showing that high levels of CCCs immunity in convalescent patients are associated with improved survival in COVID-19 patients (60, 61).

In the present study, we detected pre-existing CCCs/SARS-CoV-2 cross-reactive memory CD4^+^ and CD8^+^ T cells specific to many conserved SARS-CoV-2 epitopes in UPPHI. These results extend previous reports on the presence of specific repertoires of protective clones of memory CD4^+^ and CD8^+^ T cells in UPPHI possibly primed by previous exposure to seasonal CCCs infections and the rapid recall of α-CCCs/SARS-CoV-2 cross-reactive memory CD4^+^ and CD8^+^ T cells (1, 21, 41, 43, 62–66). UPPHI likely have different repertoires of protective and pathogenic memory CD4^+^ and CD8^+^ T cells targeting cross-reactive CCCs/SARS-CoV-2 epitopes of structural, non-structural, and accessory protein antigens that are associated with different disease outcomes in COVID-19 patients (13, 67–69). Indeed, we discovered that concomitant SARS-CoV-2/β-CCCs species (i.e., β-CCCs-HKU1 and β-CCCs-OC43) co-infection correlated with a trend toward more severe COVID-19 disease, whereas SARS-CoV-2/α-CCCs species (i.e., α-CCCs-NL63 and mainly α-CCCs-229E) co-infection significantly correlated with less severe COVID-19 disease.

The positive correlation between functional α-CCCs/SARS-CoV-2 cross-reactive memory CD4^+^ and CD8^+^ T cells and better disease outcomes in asymptomatic COVID-19 patients supports the importance of developing CoV vaccines that cross-recognize functional α-CCCs/SARS-CoV-2 cross-reactive memory CD4^+^ and CD8^+^ T cells (70–72). Pre-existing T cells cross-recognizing conserved SARS-CoV-2 epitopes that cross-react with α-CCCs, but not β-CCCs, may be important in preventing severe COVID-19 symptoms. We are currently assessing whether candidate multi-epitope-based vaccines expressing the epitopes associated with good disease outcomes that cross-react with α-CCC species (in contrast to symptomatic epitopes that cross-react with β-CCC species) would confer cross-species protection in the HLA-A2/DR1/hACE2 triple transgenic mice.

While many SARS-CoV-2 epitopes present high identity and high similarity with the four α-CCCs and β-CCC species, they did not necessarily recall the strongest SARS-CoV-2 epitope-specific CD4^+^ and CD8^+^ T-cell responses in UPPHI. For example, the SARS-CoV-2 epitopes ORF1a_1350–1365_, S_1–13_, M_176–190_, and ORF6_12–26_ recalled strong CD4^+^ T-cell responses in UPPHI but were not identical or similar with any epitopes from the four α-CCCs and β-CCC species. The same observation applies to the CD8^+^ epitopes ORF1ab_1675–1683_, S_1000–1008_, and S_1220–1228_. This suggests that the SARS-CoV-2-specific CD4^+^ and CD8^+^ T-cell responses in UPPHI may have been induced by other non-CCC pathogens, as has been reported by (73–76). Thus, in line with previous reports, we found that not all SARS-CoV-2 T-cell epitopes cross-reacted with CCC epitopes (41, 73–77). For instance, CMV T cells cross-react with SARS-CoV-2 T cells, despite low sequence homology between the two viruses, and this may contribute to the pre-existing immunity against SARS-CoV-2 (74). This is in agreement with our finding that eight of the 27 CD8^+^ T-cell epitopes (ORF1ab_1675–1683_, ORF1ab_5470–5478_, ORF1ab_6749–6757_, S_2–10_, S_958–966_, S_1220–1228_, E_20–28_, and E_26–34_) shared highly identical sequences (%id equal to 67%–78%) with epitopes from common human pathogens (EBV, *Streptococcus pneumoniae*, *Bordetella pertussis*, and *Corynebacterium diphtheriae*) and to widely distributed BCG and DTa/wP vaccines. Six of those also shared high similarity scores (S^S^≥0.8) with epitopes from EBV, *S. pneumoniae*, *B. pertussis*, and *C. diphtheriae* and widely distributed BCG and DTa/wP vaccines. CD8^+^ T cells specific to SARS-CoV-2 epitopes that share high identity and similarity with the DTwP vaccine (but not BCG vaccine) epitopes were significantly associated with asymptomatic COVID-19 infection. The most functional CD8^+^ T cells cross-recognized SARS-CoV-2 common epitopes that are highly similar and identical to epitopes from the DTwP vaccine. These findings are consistent with a previous study that described a correlation between DTwP vaccination and fewer COVID-19 deaths (59). Overall, our findings suggest that the pre-existing SARS-CoV-2-specific CD4^+^ and CD8^+^ T-cell responses in UPPHI may be the consequence of heterologous immunity induced by CCCs (31, 44, 75, 78–82), other pathogens (77), and widely administered vaccines (BCG, DTwP) (73–76).

The present comprehensive analysis demonstrates, both *in vitro* and *ex vivo*, that unvaccinated severely ill COVID-19 patients had higher frequencies of phenotypically and functionally exhausted CCCs/SARS-CoV-2 cross-reactive CD4^+^ and CD8^+^ T cells. In contrast, higher frequencies of functional CD4^+^ and CD8^+^ T cells specific to CCCs/SARS-CoV-2 epitopes were detected in unvaccinated asymptomatic COVID-19 patients. Although older COVID-19 patients tend to be more symptomatic compared to younger COVID-19 patients, the symptomatic COVID-19 patients tend to have less functional SARS-CoV-2-specific T cells, regardless of age. Similar results were obtained when age-matched symptomatic and asymptomatic COVID-19 patients were compared, suggesting that the frequency of functional SARS-CoV-2-specific T cells is age independent (data not shown). Besides CD134 and CD137 functional markers, we recently assessed the expression of additional activation and cytotoxicity markers by CCCs/SARS-CoV-2-cross-reactive CD4^+^ and CD8^+^ T cells from COVID-19 patients and healthy individuals (1). Higher frequencies of CCCs/SARS-CoV-2-cross-reactive functional memory CD8^+^ T cells were detected in both COVID-19 patients and healthy individuals. However, we have observed that COVID-19 patients and unexposed healthy individuals exhibited a different pattern of CD8^+^ T-cell immunodominance. Unlike for CD8^+^ T cells, higher frequencies of multifunctional CCCs/SARS-CoV-2-cross-reactive memory CD4^+^ T cells, expressing CD69, CD107a/b, and TNF-α were detected in COVID-19 patients compared to healthy individuals (1). However, the association of T-cell exhaustion with symptomatic and fatal COVID-19 infections in unvaccinated patients is currently being debated (6, 83). Reports using small cohorts of patients did not identify a link between higher expression of exhaustion markers and impaired function of SARS-CoV-2-specific CD4^+^ and CD8^+^ T cells in convalescent patients (84, 85). In contrast, our study used larger cohorts of COVID-19 patients with detailed clinical differentiation of symptomatic and asymptomatic patients. Our data are consistent with previous reports in which a broad T-cell exhaustion with impaired function was found in both the peripheral compartment (PBMCs), the lungs, and the brain of symptomatic patients (15, 86–88) and increased levels of PD-1 in severe cases compared to those in non-severe cases (83, 89).

Moreover, we extended those reports by characterizing the exhausted SARS-CoV-2-specific CD4^+^ and CD8^+^ T cells co-expressing multiple markers of exhaustion, TIM3, TIGIT, and CTLA4, besides PD-1. There is no consensus on a specific combination of inhibitory molecules of clusters of exhaustion markers to conclude phenotypic and functional exhaustion of epitope-specific CD4^+^ and CD8^+^ T cells. Overall, the major markers (or pathways) described as associated with CD4^+^ and CD8^+^ T-cell exhaustion include PD-1, TIGIT, CTLA-4, and TIM-3. While various combinations of these exhaustion have been used to demonstrate T-cell exhaustion, typically the PD-1 and TIM-3 combination is mainly used to demonstrate CD4^+^ and CD8^+^ T-cell exhaustion and dysfunction. In this study, we have used the combination of PD-1 and TIGIT exhaustion markers, on the one hand, and the combination of CTLA-4 and TIM-3 exhaustion markers, on the other hand, to demonstrate that increased frequencies of phenotypically exhausted SARS-CoV-2 epitope-specific CD4^+^ and CD8^+^ T cells are associated with severe COVID-19 disease, as previously reported in other systems (90–97). These findings suggest impaired functionality in SARS-CoV-2-specific CD4^+^ and CD8^+^ T cells, along with generally lower interferon-gamma (IFN-γ) and tumor necrosis factor-alpha (TNF-α) production, is associated with symptomatic and fatal Infections in unvaccinated COVID-19 patients. Our results also agree with previous reports highlighting that a prior “original antigenic sin” (OAS) potentially linked to prior exposure to seasonal CCCs might skew CCCs/SARS-CoV-2 cross-reactive CD4^+^ and CD8^+^ T cells toward an exhausted phenotype (98). Because severely ill patients preferentially developed higher frequencies of co-infection with β-CCC species and higher frequencies of pre-existing β-CCCs/SARS-CoV-2 cross-reactive memory CD4^+^ and CD8^+^ T cells, T-cell exhaustion may be related to prior exposure to seasonal β-species of CCCs.

The present study has comprehensively characterized CCCs/SARS-CoV-2 cross-reactive memory CD4^+^ and CD8^+^ T cells in blood samples from over 140 unvaccinated symptomatic and asymptomatic COVID-19 patients. However, there remain several gaps in our understanding. First, the study of CCCs/SARS-CoV-2 cross-reactive memory CD4^+^ and CD8^+^ T cells in unvaccinated symptomatic and asymptomatic COVID-19 patients has not been adjusted retrospectively to previous CCCs infections, due to the lack of pre-COVID-19 samples. At this point, the vast majority of adults in the United States have been infected and/or received at least one dose of the COVID-19 vaccine (37, 38); thus, going forward, characterizing pre-COVID-19 cross-reactive memory CD4^+^ and CD8^+^ T cells in unvaccinated COVID 19 patients will be very difficult (39). Second, the study did not follow up with the COVID-19 patients at later times points after convalescence; hence, the reported CCCs/SARS-CoV-2 cross-reactive memory CD4^+^ and CD8^+^ T cell characteristics are reflective of their status shortly after exposure to SARS-CoV-2 or during the symptomatic disease. Although we assessed SARS-CoV-2-specific CD4^+^ and CD8^+^ T cell responses at an early stage of the disease (blood sampled on average 5 days after the appearance of the first reported symptoms), the precise timing of the patient’s first exposure to SARS-CoV-2 is not known. Third, since the T-cell responses reported in this study were assessed in the peripheral blood, this may not reflect tissue-resident CD4^+^ and CD8^+^ T cells in the lungs and the brain. The reduced number of functional CCCs/SARS-CoV-2 cross-reactive memory CD4^+^ and CD8^+^ T cells detected in the peripheral blood of symptomatic COVID-19 patients may be due to T-cell redistribution to other organs, such as the lungs and the brain. The asymptomatic infections in unvaccinated COVID-19 patients might be attributed to homing and redistribution of high numbers of functional CCCs/SARS-CoV-2 cross-reactive CD4^+^ and CD8^+^ T cells into the lungs of unvaccinated asymptomatic COVID-19 patients, rather than in peripheral blood. In this context, we recently reported that high frequencies of functional lung-resident memory CD4^+^ and CD8^+^ T cells contributed to protection against COVID-19-like symptoms and death caused by SARS-CoV-2 infection in a mouse model (2). Thus, future studies should investigate tissue-resident CD4^+^ and CD8^+^ T cells in the lungs to determine whether their frequency and function correlate with protection from symptomatic and fatal infections in unvaccinated COVID-19 patients. Finally, while the study enrolled 600 patients overall, the study compared the antigen specificity, frequency, phenotype, and function of common cold coronaviruses (CCCs) and SARS-CoV-2 cross-reactive memory CD4^+^ and CD8^+^ T cells, targeting genome-wide conserved epitopes in a cohort of 147 unvaccinated COVID-19 patients screened for two HLA types, HLA-DRB1*01:01 and HLA-A*02:01. Thus, future studies are being conducted to assess T cells from other HLA types. Nevertheless, our results are consistent with the hypothesis that the early presence of high numbers of functional α-CCCs/SARS-CoV-2 cross-reactive CD4^+^ and CD8^+^ T cells targeting multiple antigens was associated with protection from symptomatic and fatal SARS-CoV-2 infections in unvaccinated COVID-19 patients (99).

This report also confirms previous reports that (i) early and broad lymphopenia positively correlated with COVID-19 disease severity and mortality (86, 100–102); (ii) broad leukocytosis combined with T cell lymphopenia was present in severe COVID-19 patients and extended those findings by demonstrating that the observed T-cell lymphopenia was particularly prevalent for SARS-CoV-2-specific T cells (86, 100); and (iii) a significant age-dependent and comorbidity-associated susceptibility to COVID-19 disease, with patients over 60 years of age, and those with pre-existing diabetic and hypertension comorbidities being the most susceptible to severe COVID-19 disease (13, 20).

In conclusion, the present comprehensive analysis of specific and cross-reactive SARS-CoV-2 epitope-specific T cells reveals clear relationships between T-cell responses and disease outcomes in unvaccinated COVID-19 patients. Compared to severely ill COVID-19 patients and patients with fatal COVID-19 outcomes, the asymptomatic COVID-19 patients presented high rates of co-infection with the α-CCC species and more functional and less exhausted α-CCCs/SARS-CoV-2 cross-reactive memory CD4^+^ and CD8^+^ T cells, targeting structural, non-structural, and accessory proteins. The findings suggest functional, poly-antigenic α-CCCs/SARS-CoV-2 cross-reactive memory CD4^+^ and CD8^+^ T cells, induced following CCCs repetitive exposures, are contributing factors in reducing the severity of SARS-CoV-2 infection, as illustrated in **Figure 8**. Most of the >10 billion doses of first-generation COVID-19 vaccines are based on the Spike antigen alone (103, 104) and function mainly by inducing neutralizing antibodies (105). Because the Spike protein has undergone a substantial number of mutations with each successive viral variant, these first-generation subunit vaccines are susceptible to immune evasion by new variants and subvariants, such as XBB.1.5, EG.5 (Eris), and HV.1 sub-variants of Omicron (71, 72). To overcome this critical limitation, the next generation of COVID-19 vaccines should also target other highly conserved structural and non-structural SARS-CoV-2 antigens capable of inducing protection by cross-reactive CD4^+^ and CD8^+^ T cells (1, 106). Herein, the findings of this report provide a roadmap for developing next-generation α-CCCs/SARS-CoV-2 cross-reactive CD4^+^ and CD8^+^ T cell-based, multi-antigen, pan-Coronavirus vaccines capable of conferring cross-species protection.

## Data availability statement

The raw data supporting the conclusions of this article will be made available by the authors, without undue reservation.

## Ethics statement

The studies involving humans were approved by Between July 2020 to November 2023, 600 patients were enrolled at the University of California Irvine Medical Center with mild to severe COVID-19 under an approved Institutional Review Board–approved protocol (IRB#-2020-5779). Written informed consent was obtained from participants before inclusion. The studies were conducted in accordance with the local legislation and institutional requirements. The participants provided their written informed consent to participate in this study.

## Author contributions

LB: Conceptualization, Data curation, Formal analysis, Funding acquisition, Investigation, Methodology, Project administration, Resources, Supervision, Validation, Visualization, Writing – original draft, Writing – review & editing. PC: Conceptualization, Data curation, Formal analysis, Investigation, Methodology, Software, Validation, Visualization, Writing – original draft. SP: Conceptualization, Data curation, Formal analysis, Investigation, Methodology, Project administration, Software, Validation, Visualization, Writing – original draft. ND: Conceptualization, Data curation, Formal analysis, Investigation, Methodology, Writing – original draft. RS: Data curation, Formal analysis, Methodology, Project administration, Writing – original draft, Conceptualization. LZ: Data curation, Methodology, Writing – original draft. DT: Data curation, Resources, Writing – original draft. RE: Data curation, Resources, Writing – original draft. CF: Data curation, Resources, Writing – original draft. SS: Resources, Writing – original draft. LH: Resources, Writing – original draft. AN: Writing – review & editing, Writing – original draft. BK: Resources, Writing – original draft. EB: Data curation, Methodology, Writing – original draft. HV: Data curation, Writing – original draft. DG: Conceptualization, Formal analysis, Writing – review & editing. TJ: Conceptualization, Writing – original draft. JU: Conceptualization, Formal analysis, Writing – review & editing, Writing – original draft.
