# Supplementary material for: High frequencies of alpha common cold coronavirus/SARS-CoV-2 cross-reactive functional CD4+ and CD8+ memory T cells are associated with protection from symptomatic and fatal SARS-CoV-2 infections in unvaccinated COVID-19 patients
Source: Front Immunol. 2024 Mar 28;15:1343716. doi: 10.3389/fimmu.2024.1343716 (PMC11007208; doi:10.3389/fimmu.2024.1343716)
Supplement: Supplementary file 1 [file DataSheet_1.pdf]

Supplementary Material

Table of Content

Serial Number	Supplementary Material	Page
1	Supplementary Fig. S1	2
2	Supplementary Fig. S2	3
3	Supplementary Fig. S3	4
4	Supplementary Fig. S4	5
5	Supplementary Fig. S5	6
6	Supplementary Fig. S6	7
7	Supplementary Fig. S7	8
8	Supplementary Fig. S8	9
9	Supplementary Fig. S9	10

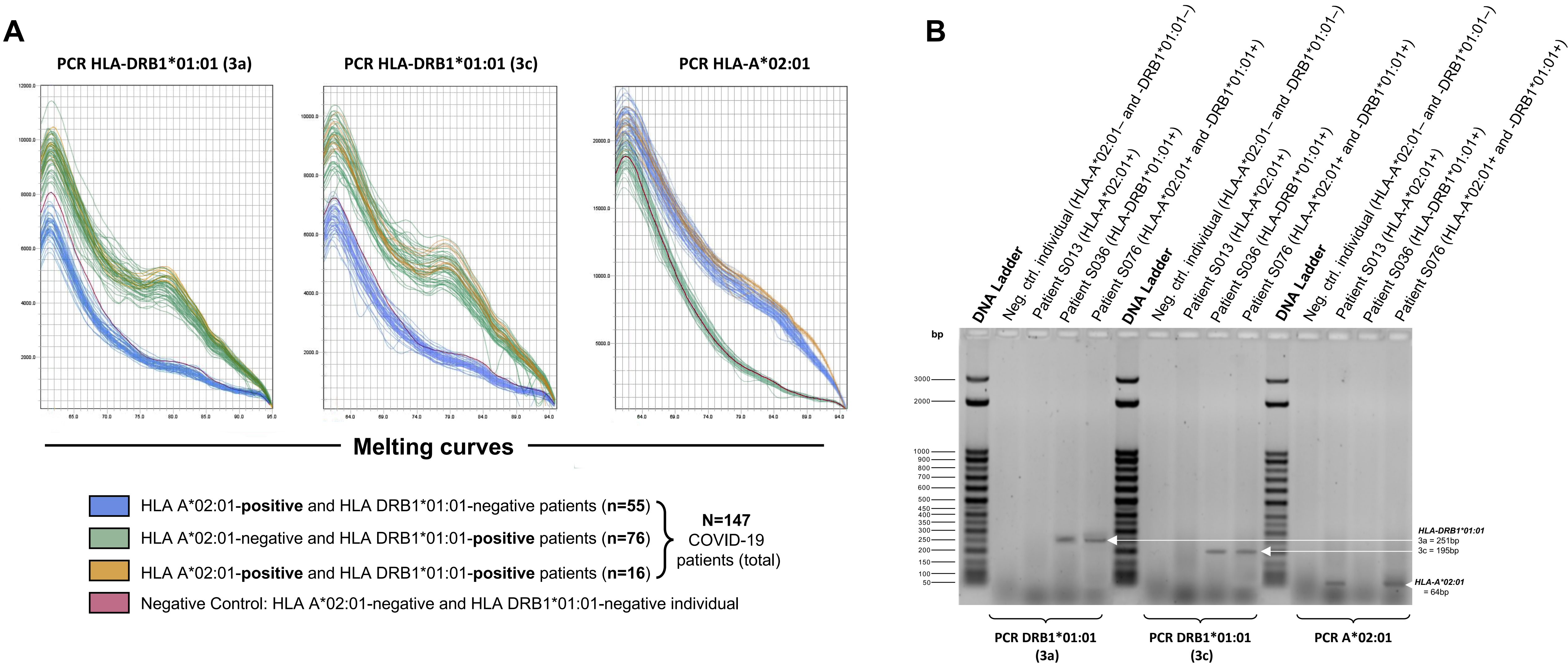

<u>Supplementary Figure S1:</u> Genotyping of HLA class-I and class-II in COVID-19 patients with various degrees of disease severity. We tested all our 600 patients for HLA-A*02:01 and HLA-DRB1*01:01 by PCRs. (**A**) Melting curves of the three PCRs performed on COVID-19 blood samples from our N=147 patients to showing their HLA-DRB1*01:01$^+$ genotype (in green – *n* = 76), their HLA-A*02:01$^+$ genotype (in blue – *n* = 55) or their HLA-DRB1*01:01$^+$ / HLA-A*02:01$^+$ genotype (in orange – *n* = 16). One double negative patient is shown as PCR negative control (in red). To determine the HLA-DRB1*01:01 genotype of a patient, two PCRs (“3a” and “3c”) were used as shown in the figure and one PCR was necessary to determine the HLA-A*02:01 genotype, as described in the Material and Methods. (**B**) Electrophoresis gel migration of the products (amplicons) of the three PCRs for the control double negative patient and for one patient HLA-A*02:01$^+$ (S013), one patient HLA-DRB1*01:01$^+$ (S036) and one double positive patient (S076).

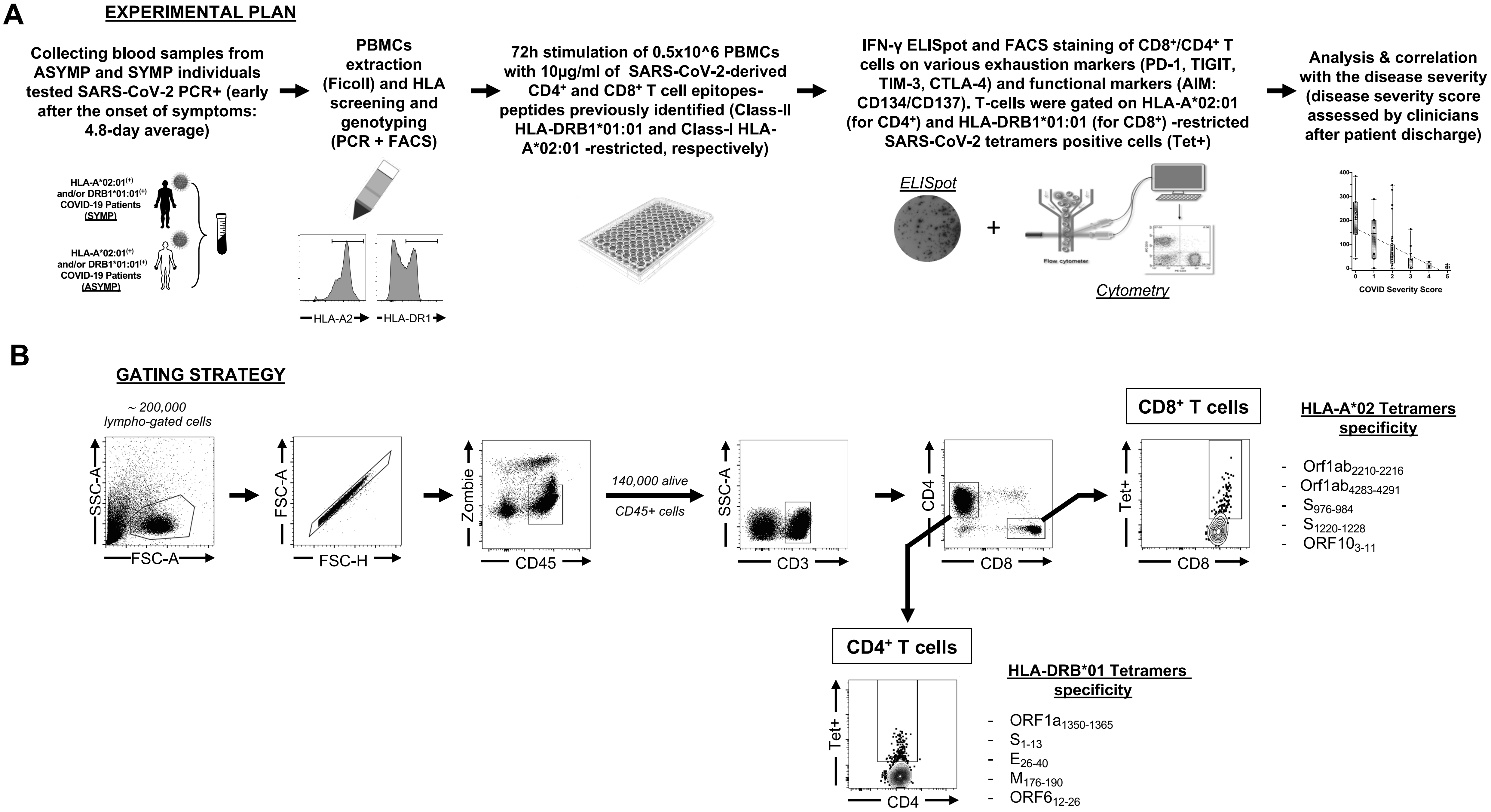

__Supplementary Figure S2__: **Experimental plan and gating strategy:** (**A**) shows experimental plan followed for the flow-cytometry experiments and the ELISpot experiments presented in the study using COVID-19 blood samples collection, patient genotyping, PBMCs extraction and peptide stimulation. (**B**) shows the gating strategy applied when analyzing the flow cytometry data presented in this study.

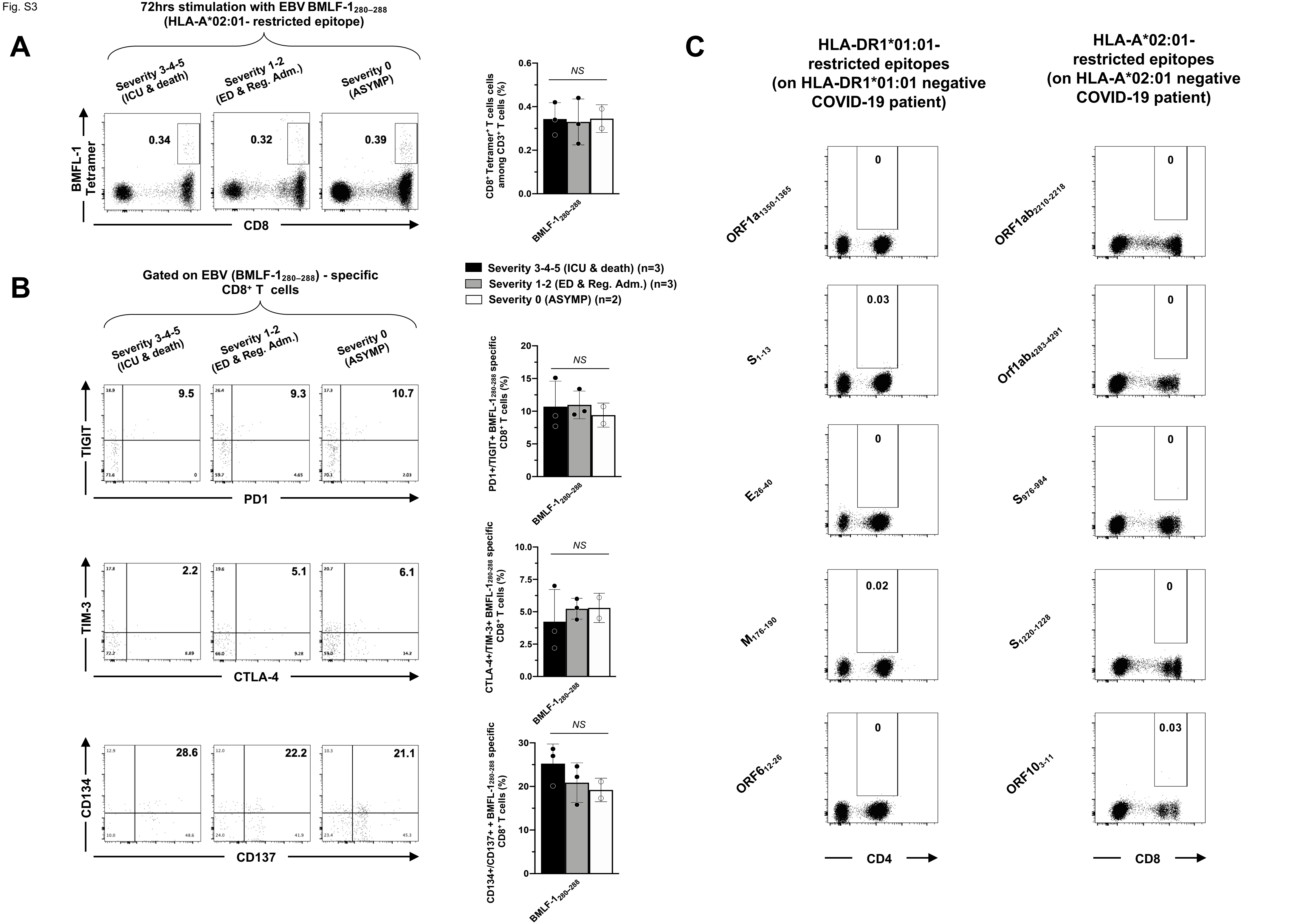

72hrs stimulation with EBV BMLF-1280-288
(HLA-A*02:01- restricted epitope)
A
Severity 3-4-5 (ICU & death)
Severity 1-2 (ED & Reg. Adm.)
Severity 0 (ASYMP)
BMFL-1 Tetramer
CD8
0.34
0.32
0.39
CD8+ Tetramer+ T cells cells among CD3+ T cells (%)
NS
BMLF-1280-288
Severity 3-4-5 (ICU & death) (n=3)
Severity 1-2 (ED & Reg. Adm.) (n=3)
Severity 0 (ASYMP) (n=2)
B
Gated on EBV (BMLF-1280-288) - specific CD8+ T cells
TIGIT
PD1
9.5
9.3
10.7
TIM-3
CTLA-4
2.2
5.1
6.1
CD134
CD137
28.6
22.2
21.1
PD1+/TIGIT+ BMFL-1280-288 specific CD8+ T cells (%)
CTLA-4+/TIM-3+ BMFL-1280-288 specific CD8+ T cells (%)
CD134+/CD137+ + BMFL-1280-288 specific CD8+ T cells (%)
C
HLA-DR1*01:01-restricted epitopes (on HLA-DR1*01:01 negative COVID-19 patient)
HLA-A*02:01-restricted epitopes (on HLA-A*02:01 negative COVID-19 patient)
ORF1a1350-1365
S1-13
E26-40
M176-190
ORF612-26
ORF1ab2210-2218
Orf1ab4283-4291
S976-984
S1220-1228
ORF103-11
0
0.03
0
0.02
0
0
0
0
0
0.03
CD4
CD8

<u>Supplementary Figure S3</u>: Frequencies of EBV (BMLF-1$_{280\text{-}288}$) specific CD8$^+$ T cells in COVID-19 patients with various degrees of disease severity and control of tetramer specificity in HLA-mismatched patients: (**A**) shows the tetramer staining against EBV BMLF-1$_{280\text{-}288}$ specific CD8$^+$ T cells after 72 hours stimulation with the corresponding peptide, in three groups of disease severity: severity 0 (ASYMP – 2 patients), severity 1-2 (mild/moderate – 3 patients) and severity 3-4-5 (severe disease – 3 patients). (**B**) Flow cytometry data showing (across the same three groups of disease severity) co-expression of the exhaustion receptors PD1, TIGIT, TIM-3 and CTLA-4 (*two upper panels*) and the expression of the AIMs CD137/CD134 (*lower panel*) in the BMLF-1$_{280\text{-}288}$ tetramers positive cell population (gated in **A**) after peptide stimulation. For both (**A** and **B**), are representative flow-cytometry dot plots (in *right panels*) and in *left panels* are the associated columns graphs with averages/means of the frequencies of the gated cells. Data are expressed as the mean $\pm$ SD. Results were considered statistically significant at $P \leq 0.05$ (one-way ANOVA). (**C**) HLA-A*02:01- or HLA-DRB1*01:01-negative COVID-19 patients used as negative controls for the different HLA-DRB1*01:01 (left) or HLA-A*02:01 (right) tetramers staining in order to assess tetramer staining specificity.

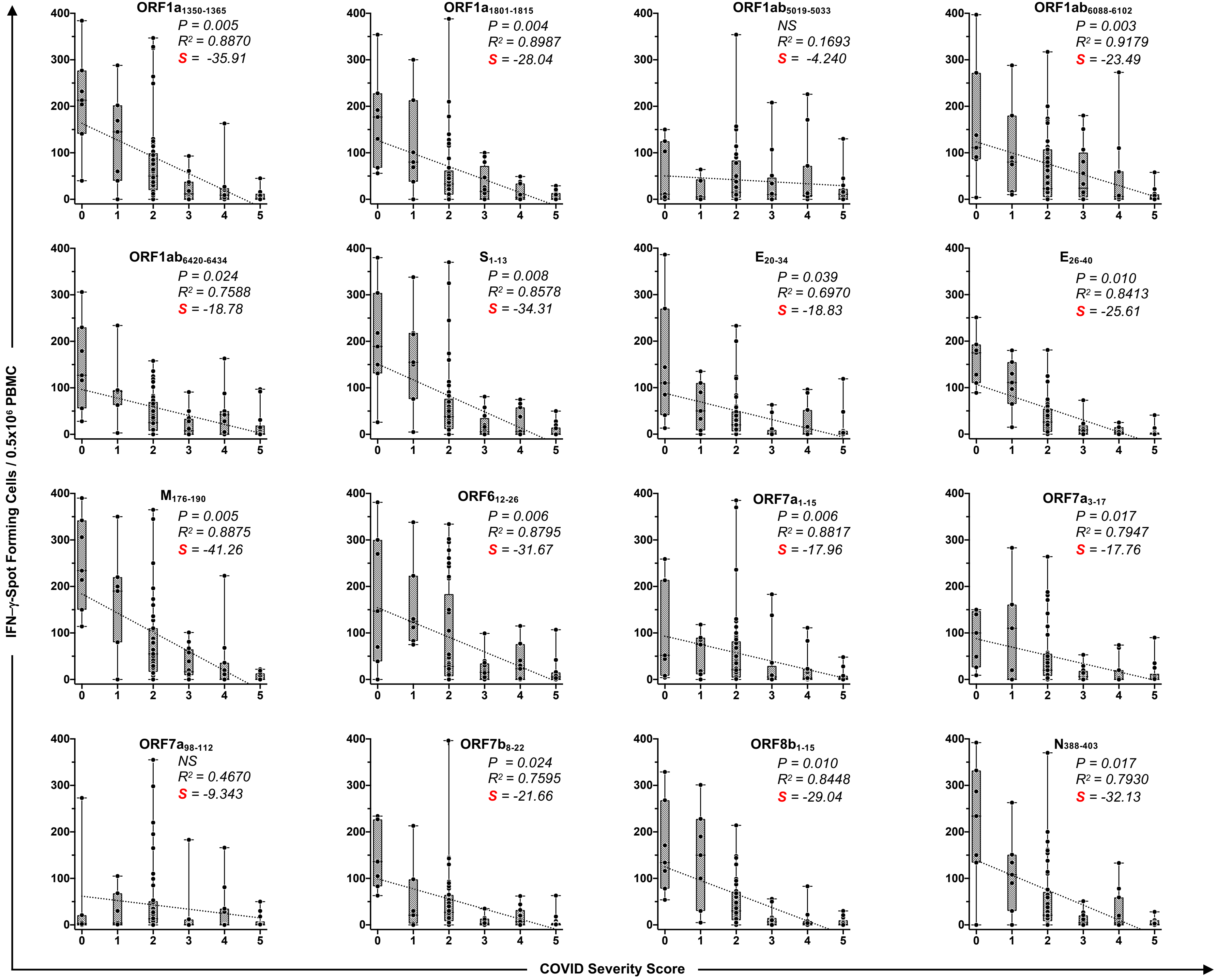
IFN–γ-Spot Forming Cells / 0.5x10^6 PBMC
COVID Severity Score
ORF1a1350-1365 P = 0.005 R² = 0.8870 S = -35.91
ORF1a1801-1815 P = 0.004 R² = 0.8987 S = -28.04
ORF1ab5019-5033 NS R² = 0.1693 S = -4.240
ORF1ab6088-6102 P = 0.003 R² = 0.9179 S = -23.49
ORF1ab6420-6434 P = 0.024 R² = 0.7588 S = -18.78
S1-13 P = 0.008 R² = 0.8578 S = -34.31
E20-34 P = 0.039 R² = 0.6970 S = -18.83
E26-40 P = 0.010 R² = 0.8413 S = -25.61
M176-190 P = 0.005 R² = 0.8875 S = -41.26
ORF612-26 P = 0.006 R² = 0.8795 S = -31.67
ORF7a1-15 P = 0.006 R² = 0.8817 S = -17.96
ORF7a3-17 P = 0.017 R² = 0.7947 S = -17.76
ORF7a98-112 NS R² = 0.4670 S = -9.343
ORF7b8-22 P = 0.024 R² = 0.7595 S = -21.66
ORF8b1-15 P = 0.010 R² = 0.8448 S = -29.04
N388-403 P = 0.017 R² = 0.7930 S = -32.13

<u>Supplementary Figure S4</u>: Magnitude of the IFN-g CD4⁺ T cell responses specific to 16 conserved SARS-CoV-2-derived epitopes in COVID-19 patients with various degrees of disease severity: Each graph named for each peptide/epitope-stimulation represent the correlations between the overall number of the SARS-CoV-2-specific IFN-g-producing $CD4^+$ T cells and the corresponding COVID-19 disease severity. For all graphs are indicated: the coefficient of determination (R^2) calculated from the Pearson correlation coefficients, its associated *P*-value and the slope (S) of the best-fitted line (dotted line) calculated by linear-regression analysis. The gray-hatched boxes in the correlation graphs extend from the 25th to 75th percentiles (hinges of the plots) with the median represented as a horizontal line in each box and the extremity of the vertical bars showing the minimum and maximum values. Results were considered statistically significant at $P \leq 0.05$.

Coulon *et al* Supplementary Fig. S5

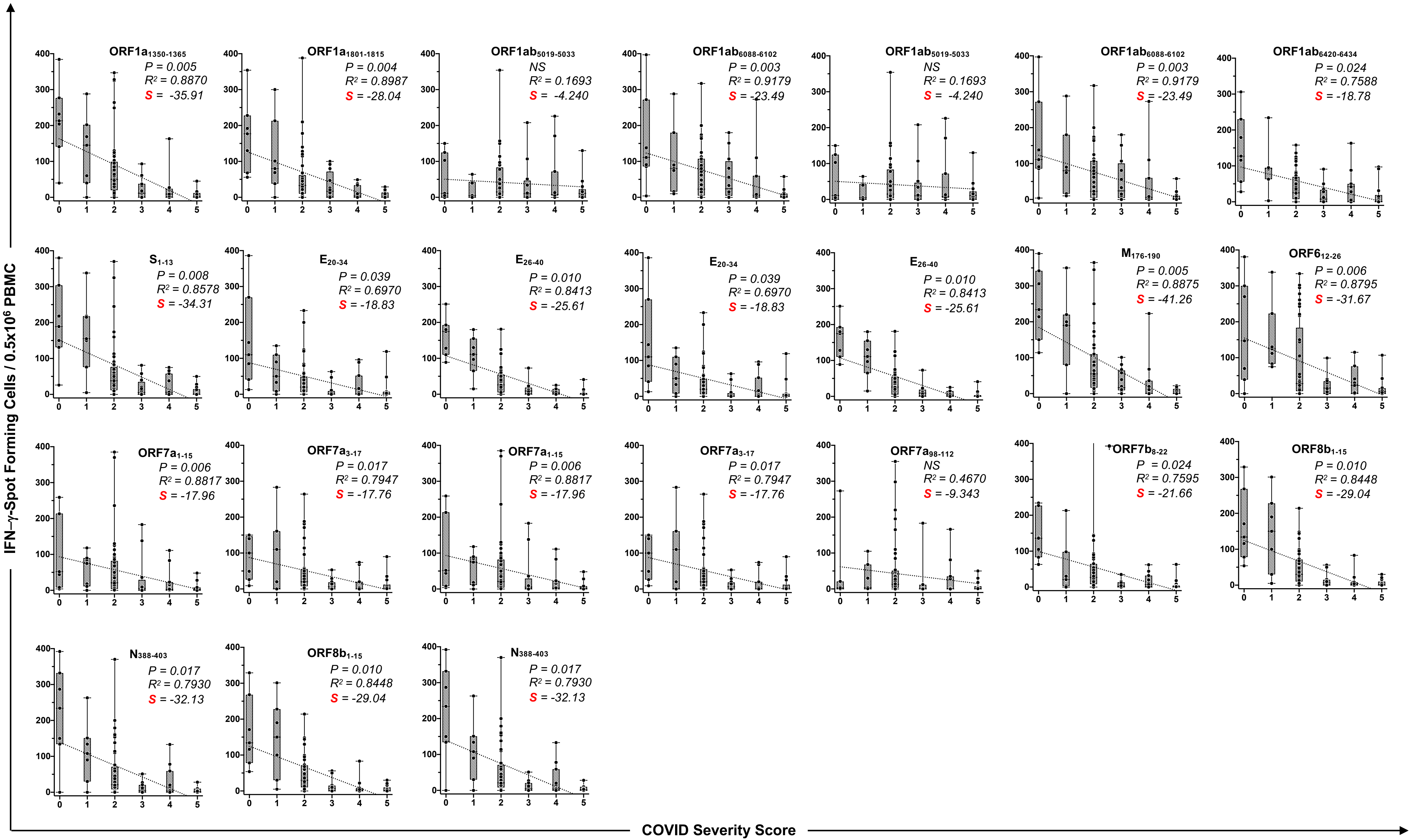

<u>Supplementary Figure S5</u>: Magnitude of the IFN-g CD8$^+$ T cell responses specific to 16 conserved SARS-CoV-2-derived epitopes in COVID-19 patients with various degrees of disease severity: Each graph named for each peptide/epitope-stimulation represent the correlations between the overall number of the SARS-CoV-2-specific IFN-g-producing CD8$^+$ T cells and the corresponding COVID-19 disease severity. For all graphs are indicated: the coefficient of determination (R^2) calculated from the Pearson correlation coefficients, its associated *P*-value and the slope (S) of the best-fitted line (dotted line) calculated by linear-regression analysis. The gray-hatched boxes in the correlation graphs extend from the 25th to 75th percentiles (hinges of the plots) with the median represented as a horizontal line in each box and the extremity of the vertical bars showing the minimum and maximum values. Results were considered statistically significant at $P \leq 0.05$.

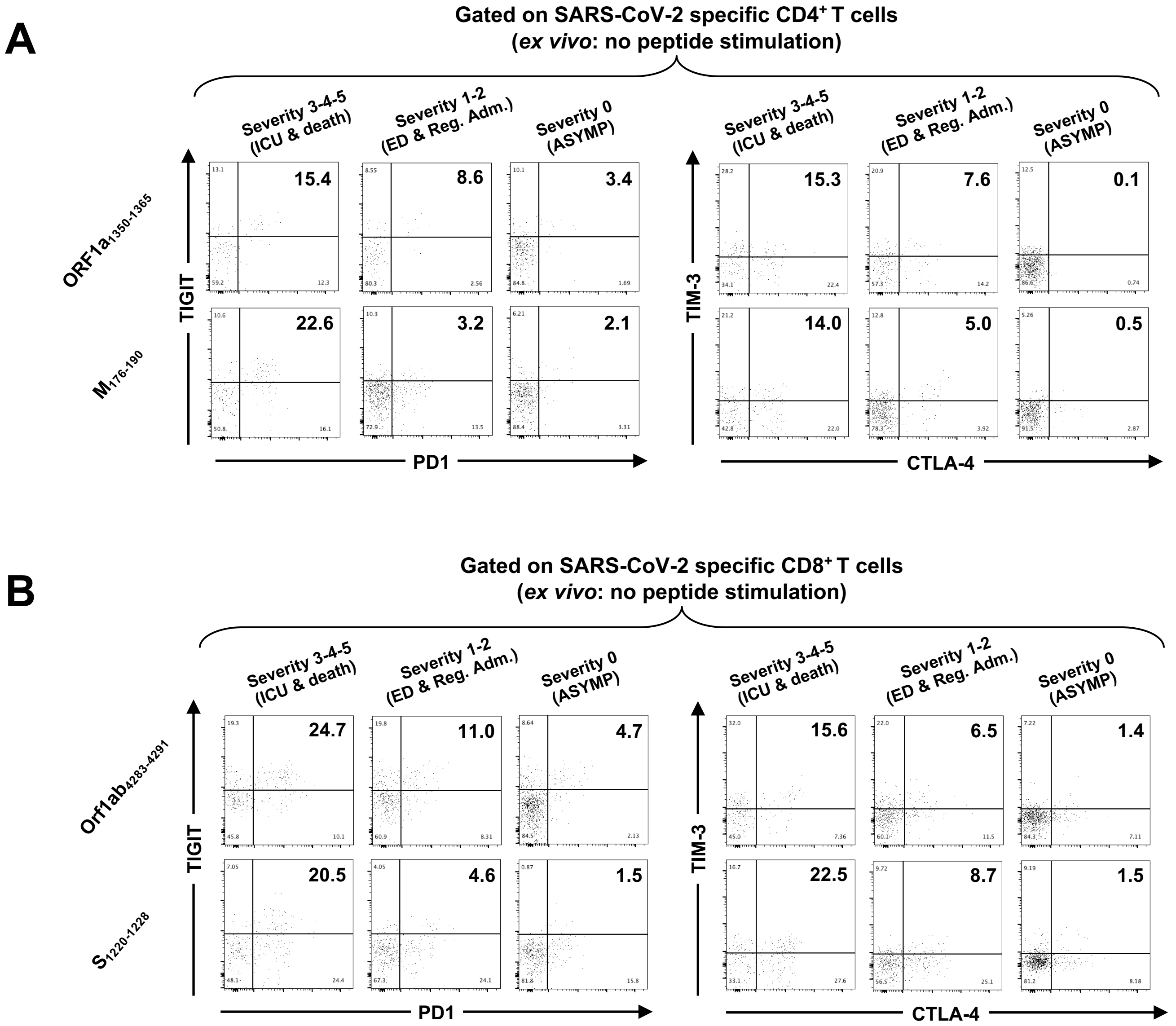

__Supplementary Figure S6__: **Measure of the CD4$^+$ and CD8$^+$ SARS-CoV-2 epitope-specific T cells exhaustion markers expression *ex vivo* in COVID-19 patients with various degrees of disease severity:** Phenotypic exhaustion markers PD1, TIGIT and TIM-3, CTLA-4 co-expression were measured by flow cytometry on the tetramer-positive SARS-CoV-2 specific CD4$^+$ (**A**) and CD8$^+$ (**B**) T cells in absence of any peptide stimulation.

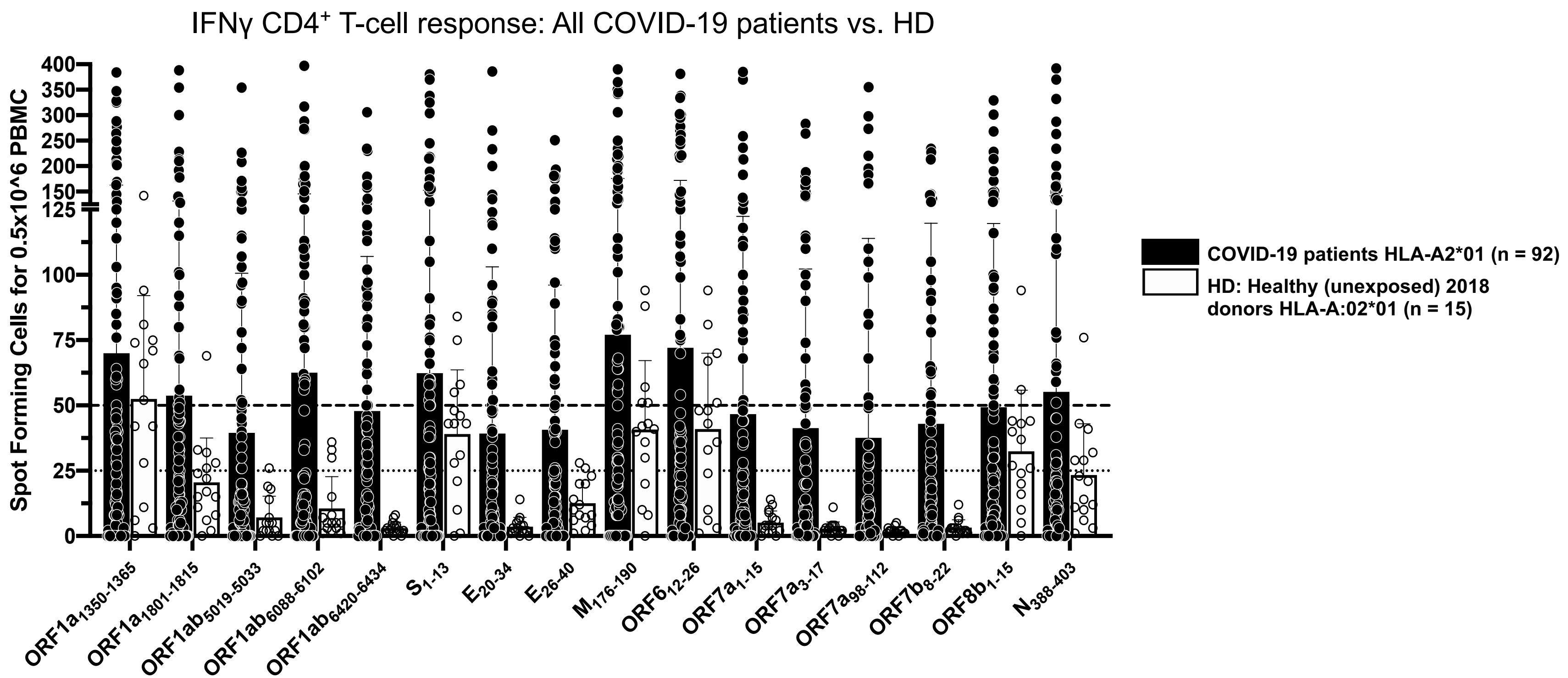
IFNγ CD4+ T-cell response: All COVID-19 patients vs. HD
Spot Forming Cells for 0.5x10^6 PBMC
400
350
300
250
200
150
125
100
75
50
25
0
ORF1a1350-1365
ORF1a1801-1815
ORF1ab5019-5033
ORF1ab6088-6102
ORF1ab6420-6434
S1-13
E20-34
E26-40
M176-190
ORF612-26
ORF7a1-15
ORF7a3-17
ORF7a98-112
ORF7b8-22
ORF8b1-15
N388-403
COVID-19 patients HLA-A2*01 (n = 92)
HD: Healthy (unexposed) 2018 donors HLA-A:02*01 (n = 15)

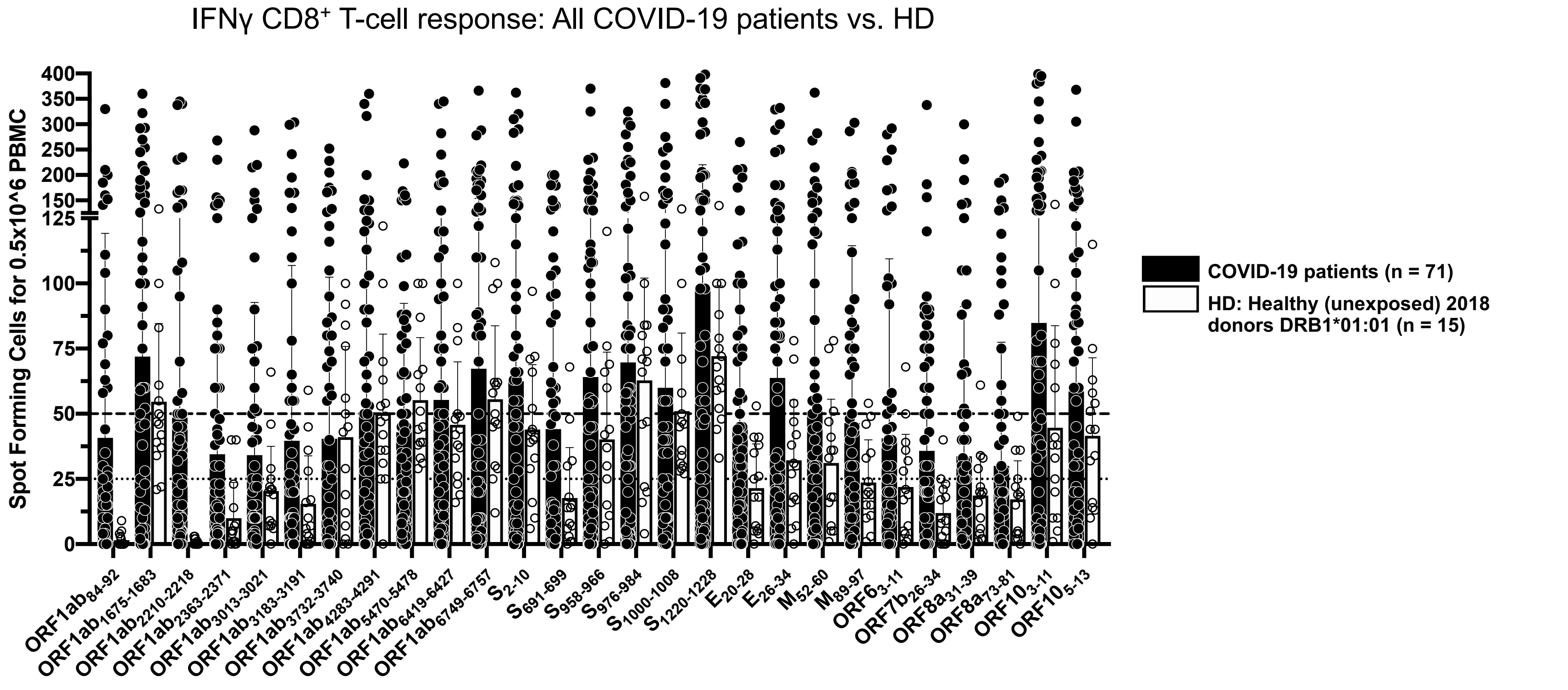
IFNγ CD8+ T-cell response: All COVID-19 patients vs. HD
Spot Forming Cells for 0.5x10^6 PBMC
400
350
300
250
200
150
125
100
75
50
25
0
ORF1ab84-92
ORF1ab1675-1683
ORF1ab2210-2218
ORF1ab2363-2371
ORF1ab3013-3021
ORF1ab3183-3191
ORF1ab3732-3740
ORF1ab4283-4291
ORF1ab5470-5478
ORF1ab6419-6427
ORF1ab6749-6757
S2-10
S691-699
S958-966
S976-984
S1000-1008
S1220-1228
E20-28
E26-34
M52-60
M89-97
ORF63-11
ORF7b26-34
ORF8a31-39
ORF8a73-81
ORF103-11
ORF105-13
COVID-19 patients (n = 71)
HD: Healthy (unexposed) 2018 donors DRB1*01:01 (n = 15)

__Supplementary Figure S7__: **CD4⁺ and CD8⁺ T cell responses specific to SL-CoVs-conserved SARS-CoV-2-derived epitopes, detected in all COVID-19 patients (regardless of disease severity) and in unexposed Healthy individuals:** Both graphs show IFN-g ELISpot data from COVID-19 patients without disease categories breakdown, compared with ELISpot data from unexposed healthy individuals (HD). The *Upper graph* (related to **Fig. 1**) shows average SFCs after 72 hours CD4-peptide stimulation of COVID-19+ HLA-A*02:01^{+} patients' PBMCs (n = 71; black bars: SARS-CoV-2 specific CD4^{+} T cell response) or of HD' PBMCs (n = 15; white bars: SARS-CoV-2 cross-reactive CD4^{+} T cell response). Likewise, the *lower graph* (related to **Fig. 2**) shows average SFCs after 72 hours CD8-peptide stimulation of COVID-19+ HLA-DRB1*01:01^{+} patients' PBMCs (n = 92; black bars: SARS-CoV-2 specific CD8^{+} T cell response) or of HD' PBMCs (n = 15; white bars: SARS-CoV-2 cross-reactive CD8^{+} T cell response). A mean SFCs between 25 and 50 correspond to a medium/intermediate response whereas a strong response is defined for a mean SFCs > 50 per 0.5×10^6 stimulated PBMCs.

Coulon *et al* Supplementary Fig. S8

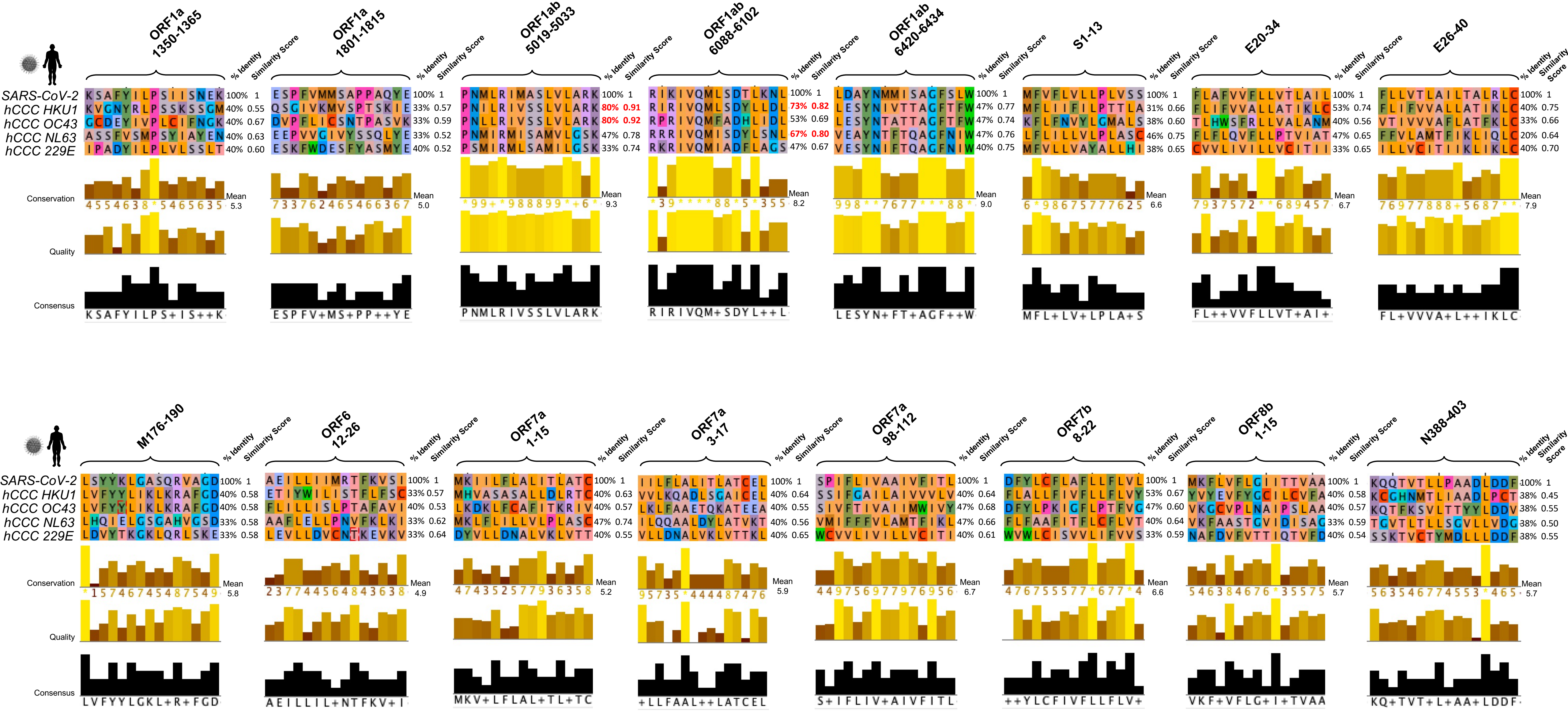

Supplementary Figure S8: Best matching sequences of CCCs epitopes with 16 $CD4^+$ SARS-CoV-2-derived epitopes: Matching CCCs peptides were chosen after combining both MSA and ECT analysis (see Materials and Methods). Each panel in both figures represent the alignment of one SARS-CoV-2 epitope and the four corresponding CCCs best matching peptide sequences. SARS-CoV-2 peptide sequence is set as 100% identity. The Amino Acids color-code was generated with Gecos software (https://gecos.biotite-python.org) using the following parameters: gecos --matrix BLOSUM62 --lmin 60 --lmax 75 –f. As a result, the distance between two Amino Acids in the substitution matrix (BLOSUM62) corresponds to the perceptual visual differences in the color scheme. Similarity score (S^S) based on such matrix are a good predictive measure of potential cross-reactivity (along with % of peptide identity). $S^S \geq 0.80$ and %id ≥ 67% are in red. Identity percentages, Similarity scores, conservation and consensus sequences are indicated in both figures for each panel.

Coulon et al Supplementary Fig. S9

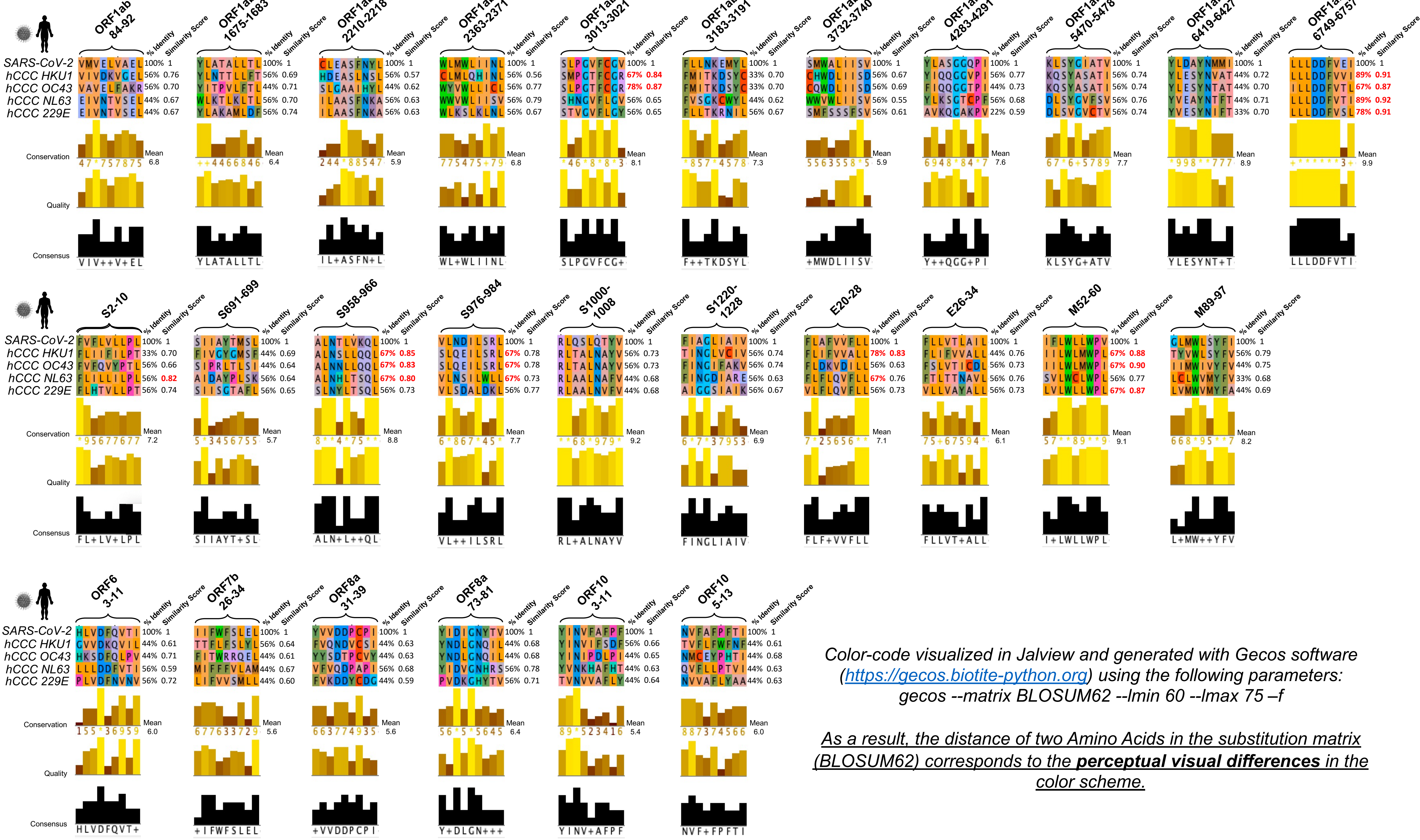

Color-code visualized in Jalview and generated with Gecos software (https://gecos.biotite-python.org) using the following parameters: gecos --matrix BLOSUM62 --lmin 60 --lmax 75 –f

*As a result, the distance of two Amino Acids in the substitution matrix (BLOSUM62) corresponds to the **perceptual visual differences** in the color scheme.*

<u>Supplementary Figure S9</u>: Best matching sequences of CCCs epitopes with 27 CD8⁺ SARS-CoV-2-derived epitopes: Matching CCCs peptides were chosen after combining both MSA and ECT analysis (see Materials and Methods). Each panel in both figures represent the alignment of one SARS-CoV-2 epitope and the four corresponding CCCs best matching peptide sequences. SARS-CoV-2 peptide sequence is set as 100% identity. The Amino Acids color-code was generated with Gecos software (https://gecos.biotite-python.org) using the following parameters: gecos --matrix BLOSUM62 --lmin 60 --lmax 75 –f. As a result, the distance between two Amino Acids in the substitution matrix (BLOSUM62) corresponds to the perceptual visual differences in the color scheme. Similarity score (S^S) based on such matrix are a good predictive measure of potential cross-reactivity (along with % of peptide identity). S^S ≥ 0.80 and %id ≥ 67% are in red. Identity percentages, Similarity scores, conservation and consensus sequences are indicated in both figures for each panel.
